# Human immunodeficiency virus type-1 (HIV-1) evades antibody-dependent phagocytosis

**DOI:** 10.1371/journal.ppat.1006793

**Published:** 2017-12-27

**Authors:** Johannes S. Gach, Margaux Bouzin, Marcus P. Wong, Veronika Chromikova, Andrea Gorlani, Kuan-Ting Yu, Brijesh Sharma, Enrico Gratton, Donald N. Forthal

**Affiliations:** 1 Department of Medicine, Division of Infectious Diseases, University of California, Irvine School of Medicine, Irvine, California, United States of America; 2 Department of Biomedical Engineering, University of California, Irvine, Irvine, California, United States of America; 3 Laboratory for Fluorescence Dynamics, University of California, Irvine, Irvine, California, United States of America; 4 Department of Microbiology, Icahn School of Medicine at Mount Sinai, New York, New York, United States of America; 5 Department of Molecular Biology and Biochemistry, University of California, Irvine, Irvine, California, United States of America; University of Zurich, SWITZERLAND

## Abstract

Fc gamma receptor (FcyR)-mediated antibody functions play a crucial role in preventing HIV infection. One such function, antibody-dependent phagocytosis (ADP), is thought to be involved in controlling other viral infections, but its role in HIV infection is unknown. We measured the ability of HIV-specific polyclonal and monoclonal antibodies (mAbs) to mediate the internalization of HIV-1 virions and HIV-1-decorated cells by phagocytes. To measure ADP of virions, we primarily used a green-fluorescent protein-expressing molecular clone of HIV-1_JRFL_, an R5, clinical isolate, in combination with polyclonal HIVIG or mAbs known to capture and/or neutralize HIV-1. THP-1 and U937 cells, as well as freshly isolated primary monocytes from healthy individuals, were used as phagocytic effector cells, and uptake of virions was measured by cytometry. We surprisingly found minimal or no ADP of virions with any of the antibodies. However, after coating virions with gp41 or with gp41-derived peptides, gp41- (but not gp120-) specific mAbs efficiently mediated phagocytosis. We estimated that a minimum of a few hundred gp41 molecules were needed for successful phagocytosis, which is similar to the number of envelope spikes on viruses that are readily phagocytosed (e.g. influenza virus). Furthermore, by employing fluorescence correlation spectroscopy, a well-established technique to measure particle sizes and aggregation phenomena, we found a clear association between virus aggregation and ADP. In contrast to virions themselves, virion-decorated cells were targets for ADP or trogocytosis in the presence of HIV-specific antibodies. Our findings indicate that ADP of virions may not play a role in preventing HIV infection, likely due to the paucity of trimers and the consequent inability of virion-bound antibody to cross-link FcyRs on phagocytes. However, ADP or trogocytosis could play a role in clearing HIV-infected cells and cells on the verge of infection.

## Author summary

Antibody-dependent phagocytosis (ADP) of virus particles is thought to play a role in clearing some viral infections. However, it is unclear what role, if any, ADP plays in preventing or controlling HIV-1 infection. We sought to directly determine if antibodies could mediate the internalization of HIV-1 into phagocytes. We found that despite the ability of tested antibodies to bind to and at times neutralize the infectivity of HIV-1, they were very inefficient at internalizing the virus into monocytes and other phagocytic cells. We further demonstrated that poor ADP activity was due to the limited number of envelope spikes found on the surface of HIV-1. Related to the low number of spikes, antibodies were inefficient at aggregating the virus, a phenomenon that was key for effective phagocytosis. On the other hand, antibodies were capable of either shearing the membrane of or internalizing cells that were coated with HIV-1. Our results cast doubt on a key role for ADP in preventing HIV-1 infection and suggest that other antibody functions are more important.

## Introduction

*In vivo* studies have demonstrated an important role for Fcγ receptor (FcγR)-mediated antibody functions in preventing lentivirus infections [1, 2]. Although correlative studies have focused primarily on antibody-dependent cellular cytotoxicity (ADCC) [3–6], there is no direct evidence to support its role or that of any other particular FcγR-mediated function in protection.

In the setting of viral infections, antibody-dependent phagocytosis (ADP) occurs when antibody-opsonized virions or cells bearing viral antigens are internalized via FcγRs into phagocytic cells such as monocytes, macrophages, neutrophils or dendritic cells [7, 8]. A related phenomenon known as trogocytosis occurs when membrane components from one cell are transferred to another, a process that can be mediated by antibody in an FcγR-dependent manner [7, 9–12]. Although possibly leading to infection of the phagocyte itself, localization of internalized cells or virions into phagolysosomes would likely result in virus deactivation. Thus, phagocytosis or trogocytosis could be an effective mechanism for antibody-mediated clearance of infectious material. Indeed, *in vivo* studies have suggested that phagocytosis plays a key role in antibody-mediated protection against some viral pathogens, including influenza virus [13, 14] and West Nile virus [15]. On the other hand, internalization of antibody-opsonized dengue virus through FcγRs enhances infection of phagocytes and is thought to be a mechanism underlying severe dengue virus infections in humans [16].

Several studies have begun to explore the potential role of phagocytosis in HIV infection. These studies have relied on *in vitro* assays using FITC-labeled virus [17], fluorescent beads coated with Env glycoprotein [18], or virus engineered to express mCherry [19]. Nonetheless, with respect to HIV, simian immunodeficiency virus (SIV) and chimeric HIV/SIV (SHIV), the role of phagocytosis in preventing or enhancing infection remains unknown.

In this study, we sought to measure HIV-specific ADP with an eye toward establishing a biologically relevant assay that could be used to investigate correlates of protection during vaccine trials. To our surprise, we found little or no ADP of virions, even when opsonized with broadly neutralizing antibodies that avidly capture infectious virus. Moreover, our results suggest that the paucity of envelope spikes on the surface of HIV-1 virions results in a level or orientation of bound antibody that is likely inadequate for FcγR crosslinking. On the other hand, CD4 lymphocytes adsorbed with HIV-1 virions are a target for phagocytosis or trogocytosis.

## Materials and methods

### Ethical statement

Peripheral blood from anonymous healthy donors was obtained expressly for this research (through the University of California, Irvine Normal Blood Donors Program) with informed, written consent in accordance with the Institutional Review Board at the University of California, Irvine.

### Reagents

The following reagents were obtained through the NIH AIDS Reagent Program, Division of AIDS, NIAID, NIH: HIV-1_MN_-gp41, HIV Gag-iGFP_JRFL, HIV Gag-iGFP_NL4-3, 2G12, VRC01, F425 B4e8, PG9, PG16, 2F5, 10E8, 4E10, Z13e1, HIVIG, and D50. The HIV-1 gp120-specific antibody b12 and the control antibody DEN3 (anti-dengue NSI human IgG1 [20]) were generous gifts of Dr. Dennis Burton. The MPER peptide 09129 was kindly provided by Dr. Michael Zwick. The phosphatidylserine-targeting human monoclonal antibody PGN635 [21] was provided by Dr. Cyril Empig (Peregrine Pharmaceuticals Inc.). Antibody 1F7 [22] was provided by Dietmar Katinger (Polymun, Austria)

### Cell lines

TZM-bl (8129) and THP-1 cells (9949) were obtained from the NIH AIDS Research and Reference Reagent program. 293T (CRL-3216) and U937 (CRL-1593.2) cells were obtained from the American Type Culture Collection (ATCC). TZM-bl and 293T cells were maintained in Dulbecco’s modified Eagle’s medium (DMEM) supplemented with 10% heat-inactivated fetal bovine serum (FBS) (Atlas). THP-1 and U937 cells were cultivated in RPMI-1060 medium supplemented with 10% heat-inactivated FBS. All media contained a mixture of 0.1 mg/mL of penicillin and streptomycin (Gibco).

### Virus production

Full-length molecular clones of HIV-1_SF162_, HIV-1_JR-FL_, HIV-1_JRCSF_, HIV-1_HxB2_, HIV-1_iGFP/JR-FL,_ and HIV-1_iGFP/NL4-3_ were generated by transfecting 1 x 10^7^ 293T cells with 20 μg of the HIV-1 plasmids pLAI-SF162, pLAI-JR-FL, pLAI-JRCSF, pLAI-HxB2, HIV Gag-iGFP_JRFL), and HIV Gag-iGFP_NL4-3 using polyethyleneimine (PEI) as a transfection reagent (DNA/PEI ratio = 1:3). HIV-1_VSV-g_-pseudotyped virions were generated by transfecting 1 x 10^7^ 293T cells with 40 μg of the HIV backbone plasmid pSG3ΔEnv and 10 μg of VSV-g at the same DNA/PEI ratio as described above. After 72 hours, cell culture supernatant fluid was harvested, cleared by centrifugation at 4000 rpm for 10 min, concentrated with Lenti-X concentrator (Clontech) according to the manufacturer’s instructions, and finally stored at -80°C. The primary isolate HIV-1_JR-FL_ was produced in phytohemagglutinin (PHA)-stimulated peripheral blood mononuclear cells (PBMCs) from healthy donors and stored at -80°C.

### HIV-1 p24 enzyme linked immunoassay (ELISA)

The HIV-1 p24 content of all viral preparations was determined using the RETRO-TEK HIV-1 p24 antigen ELISA kit (ZeptoMetrix) following the manufacturer’s instructions. Levels of p24 were calculated by a point-to-point algorithm.

### Virus infectivity assay

To determine infectious units per mL, HIV-1 virions were titered on TZM-bl reporter cells at 1×10^4^ cells per well in a final concentration of 0.01 mg/mL DEAE-dextran and incubated for 48 hours at 37°C. Cells were then washed with PBS, lysed, and finally developed with luciferase assay reagent (Promega) according to the manufacturer’s instructions. Luminescence in relative light units (RLU) was measured using a Synergy 2 microplate luminometer (BioTek).

### Virus capture assay

Ninety-six well plates (Corning) were coated with 250 ng (0.005 mg/mL) of rabbit anti-human IgG gamma chain-specific antibody (Rockland) per well and incubated over night at 4°C. Plates were then washed three times with PBS and blocked with 5% non-fat dry milk in PBS at 37°C. After one hour, plates were washed again three times and incubated with 100 ng (0.002 mg/mL) of HIV-1-specific antibodies for one hour at 37°C. After three washes, HIV-1 virions were diluted in DMEM growth medium and transferred to wells (10 ng p24/well). After 4-hours incubation at 37°C, plates were washed five times with PBS. In a final step, the infectivity of captured virions was determined using TZM-bl reporter cells as described above, or the p24 content captured on the plate was directly measured with a p24 ELISA assay. Samples were analyzed in triplicate, and assays were repeated three times.

### HIV-1 antigen depletion assay

HIV-1_iGFP/JR-FL_ virus preparations (50 ng p24/reaction) were incubated with 10 μM HIV-1_MN_ gp41 recombinant protein at 37°C. After one hour, virions were pelleted (60 min at 14,000 rpm and 4°C), and the supernatant was carefully removed and analyzed for unbound HIV-1_MN_ gp41 protein.

### ELISA for antigen depletion assay

ELISA plates were coated over night at 4°C with 0.005 mg/mL (250 ng per well) of the gp41-specific mouse monoclonal antibody D50. Samples consisting of supernatant fluid from gp41-decorated virus and a gp41 standard were serially diluted 1:3 and added to the blocked (5% non-fat dry milk in PBS containing 0.05% Tween-20) wells. Samples and the gp41 standard (starting at 0.003 mg/mL) were incubated for one hour at 37°C. Unbound proteins were washed away and plates were further incubated with the gp41-specific human monoclonal antibody Z13e1 (0.001 mg/mL). After one hour, plates were washed and incubated with an HRP-labeled anti-human Fab-specific antibody. Plates were then incubated for an additional hour at 37°C, washed, developed (TMB), stopped, and read according to the manufacturer’s instructions (Invitrogen). Samples were analyzed in triplicate, and experiments were performed at least twice.

### HIV-1 antigen ELISA

ELISA plates were coated with either D50 antibody (250 ng/well) or NeutrAvidin protein (250 ng/well). The washed and blocked plates (5% non-fat dry milk in PBS containing 0.05% Tween-20) were further incubated with 0.001 mg/mL of HIV-1_MN_ gp41 (D50 antibody plate) or 0.003 mg/mL of the MPER mimetic bio-09129 (NeutrAvidin plate). After one-hour incubation at 37°C, serial dilutions (1:5) of HIV-1-specific antibodies starting at 0.05 mg/mL were added to the plates. Antibodies were incubated for one hour at 37°C, and HIV-1 antigen-specific antibodies were detected with an HRP-labeled anti-human Fab-specific antibody. Ultimately, plates were read as described above. Samples were analyzed in duplicate, and experiments were performed at least twice.

### HIV-1 phagocytosis assay

For most experiments, HIV-1_iGFP_ virions (50 ng p24/reaction) were used. When indicated in the text, the virions were decorated with gp41 or with gp41-derived peptide (10 and 50 μM, respectively) for one hour at 37°C. Virions that did not express iGFP were also used in some experiments after staining with the lipophilic membrane dye DiO (Molecular Probes) for 20 min at 37°C. In all cases, virions were pelleted (60 min at 14,000 rpm and 4°C), and after removal of the supernatant fluid, the pellets were resuspended in RPMI-1640 medium and mixed in a 1:1 ratio with monoclonal (0.1 mg/mL) and polyclonal antibody preparations (0.4 mg/mL). The virus/antibody mixture was incubated for one hour at 37°C followed by an 80-min incubation step at 37°C with phagocytes (THP-1 cells, U937 cells or primary monocytes; 100,000 cells per well). After phagocytosis, cells were washed three times with PBS and incubated with Accutase (Innovative Cell Technologies) for 10 min at 37°C. In a final step, cells were washed once in Accutase and fixed in 4% PFA and analyzed by flow cytometry on a BD Accuri C6 (BD Biosciences) or NovoCyte flow cytometer (ACEA) for internalized virions (S1 Fig). A phagocytic score was determined by gating the samples on events representing cells and calculated as follows: Percent GFP positive × median fluorescence intensity (MFI) GFP positive. Samples were considered positive if the phagocytic score was higher than the no antibody control plus two standard deviations. Accutase treatment, presumably by more efficiently removing surface-bound virions than PBS washes alone, resulted in about a 25% reduction in the apparent phagocytic score of the positive control. Phagocytosis experiments for each virus were performed at least three times with antibody samples being tested in triplicate. The phagocytosis assay is similar to that used by Tay et al. with notable changes being the use of different viruses expressing a different fluorophore and the lack of a spinoculation step [19].

An alternative phagocytosis assay was also used that did not rely on detecting fluorophores or membrane dyes. Human PBMC-grown HIV-1_JR-FL_ (20 ng of p24) was incubated with antibodies (mAbs at 0.02 mg/mL or HIVIG or IVIG at 0.08 mg/mL) and then with U937 cells (100,000 cells per well) under the same conditions described above. After washing and Accutase treatment as above, the U937 cells were lysed in 0.2 mL of RNA lysis buffer. Viral RNA was then extracted from a fraction of the cells (25,000 cells) according to the manufacturer’s instructions (ZR Viral RNA Kit, Zymo Research) and further analyzed for internalized virus using quantitative one-step real-time RT-PCR (QuantiTect SYBR Green RT-PCR kit, Qiagen GmbH). The following HIV-1 gag primers were used: SK462 d(AGTTGGAGGA-CATCAAGCAGCCATGCAAAT); and SK431 d(TGCTATGTCAGTTCCCCTTGGTTCTCT) (AnaSpec Inc.).

A similar PCR-based assay was used to measure internalization of influenza virus. 0.025 mL/well of influenza virus strain A/Puerto Rico/8/1934 H1N1 (PR8; multiplicity of infection = 10) were incubated in U-shaped 96-well plates (Falcon) with 0.025 mL of influenza hemagglutinin-specific mAbs (FI6, 2G02, or CR9114), negative control antibody or PBS (Gibco) in triplicate for 1 hour at 37°C; the antibodies were used at a final concentration of 0.01 mg/mL. 1x10^5^ THP-1 cells were then added in a volume of 0.050 mL of RPMI/well, and the plates were incubated for another 60–80 min at 37°C. Cells were then transferred to Eppendorf tubes and washed twice with PBS. TPCK trypsin at a 1:1000 dilution in RPMI media (Gibco) was added (0.1 mL/sample), and samples were incubated at 37°C for 10 min. Following another wash with PBS, cells were resuspended in 0.1 mL of TriReagent, and RNA was extracted using the Direct-zol RNA MiniPrep Plus kit (Zymo Research) according to the manufacturer’s instructions. The SuperScript III First-Strand Synthesis SuperMix for qPCR kit (Invitrogen) was used to obtain cDNA. cDNA (1 μL at 50 ng/μL) was used as a template for each qPCR reaction. qPCR was set up in 384-well plates in triplicate for each biological sample using the LightCycler 480 SYBR Green I Master (Roche) master-mix. For virus-specific amplicons, we used PR8 M2-specific primers (5’ TATCATTGGGATCTTGCACTTGA 3’ and 5’ CCTTTCGATATTCTTCCCTCATA 3’). 18S rRNA primers were used as a control. The PCR reaction was run on a LightCycler 480 II (Roche) in two technical runs. Virus was quantified and reported as number of viral particles based on a standard curve generated by serial dilutions of virions in a plaque assay; viral RNA was extracted from the serial dilutions and transcribed as above.

### Phagocytosis of aggregated HIV-1 virions

HIV-1_iGFP/JR-FL_ virions (50 ng p24/reaction) were incubated with envelope-specific antibodies as described above. The antibody-opsonized virions were then incubated with either 0.15 mg/mL of goat anti-human Fc antibody (Jackson ImmunoResearch) or with 0.05 mg/mL of goat F(ab’)_2_ anti-human F(ab’)_2_ antibody (Jackson ImmunoResearch). Phagocytosis and subsequent evaluation of virus uptake was performed as described above.

### Cell surface-decorated HIV-1 phagocytosis assay

HIV-1_iGFP/JR-FL_ virions were pelleted for one hour at 14,000 rpm and 4°C. Next, CEM.NKr-CCR5 target cells were washed with PBS and transferred to a 96-well flat-bottom plate (5x10^5^ cells/well) and subsequently mixed with 500 ng p24 (~ 1.25 x 10^4^ virions per target cell) in a final volume of 0.1 mL and spinoculated for 2 hours at 2500 rpm and 10°C. After spinoculation, virus-coated cells were washed with ice-cold PBS and incubated with HIV-1-specific antibodies at a final concentration of 0.05 mg/mL for 45 min at 4°C. Meanwhile, THP-1 effector cells were washed and added to the opsonized CEM.NKr-CCR5 target cells at a ratio of 1:1 and incubated on a shaker for one hour at 37°C. Cells were then washed with PBS and further incubated with a PE-conjugated anti-CD32-specific antibody (BioLegends) for 30 min at room temperature. Cells were finally washed twice, fixed in 4% PFA in PBS and analyzed by flow cytometry (S1 Fig). All experiments were performed in triplicate and repeated at least four times.

### ImageStream cytometry

ImageStream cytometry analysis of HIV-1_iGFP/JR-FL_ internalization was performed as previously described [19]. In brief, after conducting the ADP assay as above, THP-1 cells were resuspended at a final concentration of 5 x 10^6^ cells/mL. Using an ImageStreamX Mark II Imaging Flow Cytometer (EMD Millipore), fluorescent-virus images were collected in channel 2 (480-560nm) at 40x magnification. Internalization of positive events was measured by applying the ImageStream IDEAS Internalization and Spot Wizard algorithms, which defined the internal area as a mask of erosion of 4 pixels into the brightfield perimeter of the cell. Cells with internalized virus were defined by two criteria: an internalization score greater than 0.3 and a Spot Value greater than 3, in order to exclude surface-bound virus and background fluorescence, respectively.

ImageStream cytometry was also used to visualize internalization of HIV-1_iGFP/JR-FL_ used to decorate the surface of CEM.NKR-CCR5 cells. THP-1 cells were surface stained with anti-CD32-PE (BioLegend), and PE+/GFP+ images were collected in channels 2 and 3 (560–595 nm). Focused, single cells (based on gating on gradient root mean square, area, and aspect ratio of the brightfield image) were analyzed.

### Fluorescence correlation spectroscopy

All fluorescence correlation spectroscopy (FCS) [23–25] experiments (see Theory Section in S1 Text) were performed on a Zeiss LSM 510 META confocal microscope equipped with a Confocor 3 unit (Zeiss, Jena, Germany). GFP excitation was primed by the 488-nm line of an air-cooled Argon laser, with a typical power of ≈10 μW on the sample plane. The emitted signal was collected in epi-fluorescence geometry by a water-immersion objective (Zeiss C-Apochromat, 40x, N.A. = 1.2) and detected by an Avalanche Photo-Diode via a 505-nm long pass filter. A 1.36-Airy-units pinhole configuration was adopted for all the experiments and disposable Lab-Tek borosilicate eight-well chambers were employed as sample holders with a typical 0.25 mL sample volume.

For each sample, three 280-second-long fluorescence intensity time traces were acquired with 5-μs temporal resolution, and a 10-second-long auto-correlation function (ACF; average ± SEM) was subsequently computed by means of SimFCS software (developed by Enrico Gratton, Laboratory for Fluorescence Dynamics). Globals for Spectroscopy (developed by Prof. Gratton) was employed for the non-linear fit of the ACFs to either a one-component or a two-component free three-dimensional Brownian diffusion model (S1 Text). The ω_0_ radial waist of the focused laser beam, which was treated as a fixed parameter for the ACFs fit, was calibrated daily by measuring the ACF of a reference sample of known diffusion coefficient freely diffusing in MilliQ water (100-nm yellow-green fluorescent beads, D = 4.34 μm^2^/s, Invitrogen, Carlsbad, CA, USA). Fluorescent bead samples were vortexed before each measurement to avoid bead aggregation, and the recovered ACFs were always fit to a single-component free 3D Brownian diffusion model (S1 Text). An example ACF is shown (S2 Fig). Recovered ω_0_ values ranged from 0.300 μm to 0.335 μm.

Confidence intervals associated with the FCS-recovered parameters were quantified by a so-called rigorous error analysis [26, 27], which is based on the exploration of the curvature of the chi-square surface of the ACF’s fit close to the surface minimum. Specifically, the chi-square surface of the fit was explored along all the parameter coordinates to determine all possible parameter combinations compatible with a predefined maximum variation (one standard deviation in the present case) of the chi-square value. Such a procedure yields, for each parameter, the standard deviation as well as its minimum and maximum values compatible with the required variation of the fit chi-square value. Standard deviations obtained by the rigorous error analysis were subsequently exploited to run a one-way ANOVA statistical analysis followed by the Tukey’s multiple-comparisons test on the FCS results.

Validation of FCS results in terms of particle size and aggregation was obtained by fluorescence confocal imaging of the same virion preparations. Provided that the fluorescence signal exclusively arises from virion particles, and provided that sub-resolved single opsonized virions appear as diffraction-limited (~200 nm) spots in confocal images, the observation of imaged particles much larger than the confocal microscope 200 nm spatial resolution unambiguously reveals aggregation in the form of virion multimers (S2 Fig).

### Statistical analysis

Normally distributed data were analyzed using one-way ANOVA followed by Dunnett’s multiple comparison correction. If data did not pass normality tests, we used Kruskal-Wallis tests followed by Dunn’s correction for multiple comparisons. Correlations were analyzed using Spearman’s rank correlation. FCS data were analyzed with one-way ANOVA followed by Tukey’s multiple-comparisons test. All statistics were performed using Graph Pad Prism 7.0.

## Results

### Env-specific antibodies do not mediate phagocytosis of HIV-1 virions

**Fig 1:**
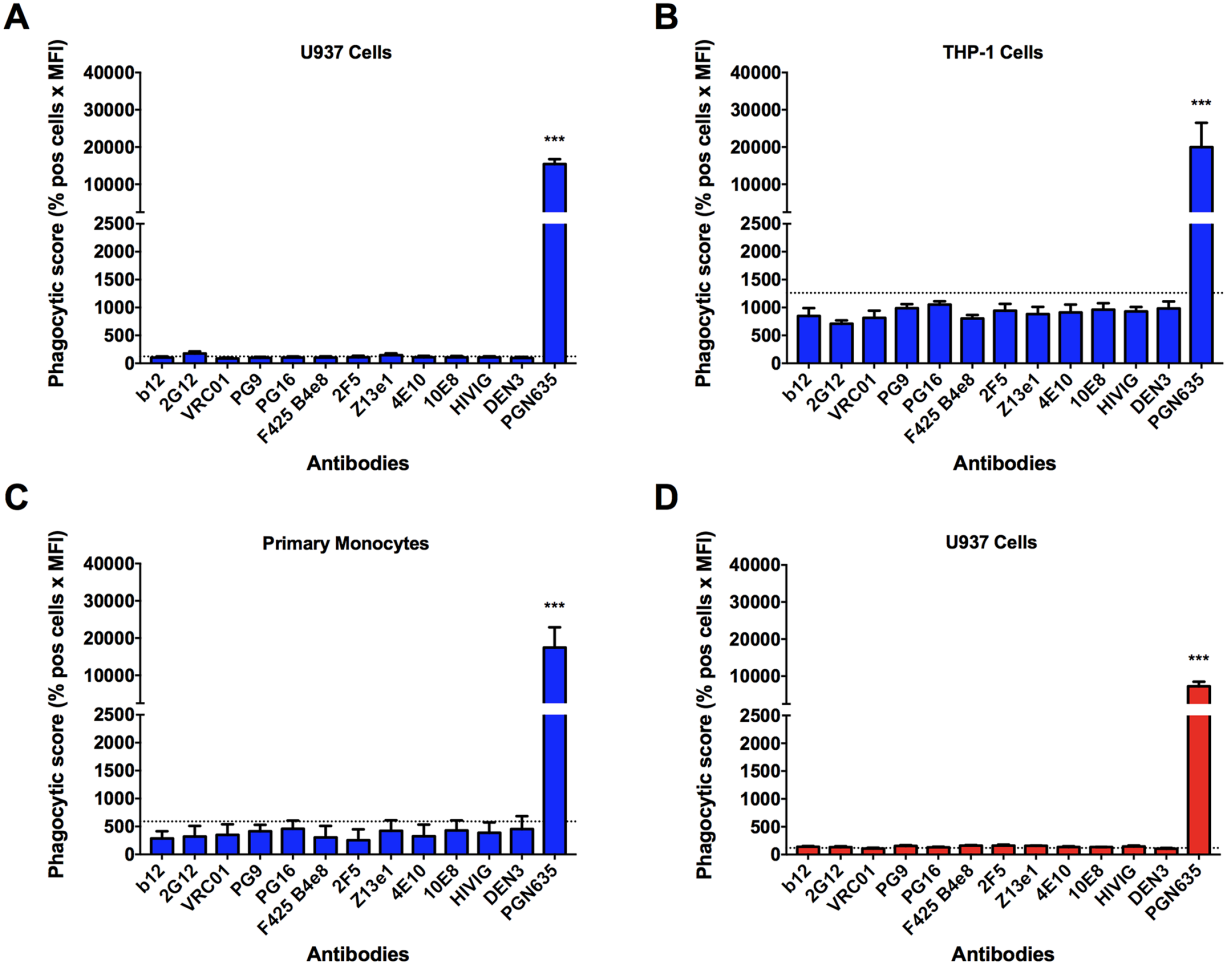
Env-specific antibodies do not mediate phagocytosis of HIV-1 virions. Internalization of HIV-1_iGFP/JR-FL_ was measured using U937 cells **(A)**, THP-1 cells **(B)**, or primary monocytes **(C)**. Uptake of HIV-1_iGFP/NL4-3_ virions was measured in U937 cells **(D)**. All mAbs were tested at a concentration of 0.05 mg/mL and the polyclonal antibody HIVIG at a concentration of 0.2 mg/mL. PGN635 is an anti-phosphatidylserine antibody included as a positive control. Data are reported as phagocytic score (% positive cells x MFI). One-way ANOVA was used in the analysis of virus uptake in the presence of an antibody compared to the no antibody control (dotted line). *P*-values are indicated with an asterisk: * *p* ≤ 0.05; ** *p* ≤ 0.01, and *** *p* ≤ 0.001. All experiments were performed in triplicate and repeated at least three times. Data are reported as means + SEM.

To investigate the role of ADP in the clearance of HIV-1 virions, we used the GFP-expressing infectious clade B isolates HIV-1_iGFP/JR-FL_ (R5 tropic) and HIV-1_iGFP/NL4-3_ (R4 tropic) and several gp120- and gp41-specific antibodies (Table 1). None of the tested antibodies were able to mediate detectable internalization of HIV-1_iGFP/JR-FL_ virions in U937 cells (Fig 1A), THP-1 cells (Fig 1B), or primary monocytes (Fig 1C) when compared to the background internalization observed in the absence of specific antibody. Since HIV-1_JR-FL_ is considered a tier 2 virus (i.e. less sensitive to neutralizing antibodies), we also tested HIV-1_iGFP/NL4-3_, a neutralization sensitive, R4 tropic, tier 1 virus. Using U937 cells, we similarly found that none of the antibodies increased the internalization of the virions above background (Fig 1D). Note that the positive control antibody PGN635, a human mAb that targets phosphatidylserine (PS) [21] resulted in substantial uptake of virions in all three phagocytic cell types (Fig 1). A F(ab’)_2_ version of PGN635 reduced the phagocytic score by an average of 88% compared with the whole mAb, thus implicating an Fc-dependent process in the phagocytosis mediated by the positive control antibody

**Table 1:** Antibodies used in ADP assays.

Epitope specificity	Antibody
CD4 binding site (CD4bs)	b12
	VRC01
	1F7
Glycan	2G12
Variable loop 1/2 glycan	PG9
	PG16
Variable loop 3	F425-B4e8
Membrane proximal external region (MPER)	2F5
	Z13e1
	4E10
	10E8
Polyclonal	HIVIG
Phosphatidylserine (PS)	PGN635

To further confirm that antibodies did not mediate phagocytosis of HIV-1 virions and to generalize our findings to HIV-1 grown in PBMCs, we used HIV-1_JRFL_ in an experimental setup similar to that used with the GFP-expressing virions. However, because of artifacts introduced by labeling virus (see below), we determined the amount of internalized virus using RT-PCR of U937 cell lysates. Again, none of the mAbs or HIVIG resulted in virus internalization above the background with no antibody or with negative control antibodies. The positive control (anti-PS) antibody, on the other hand, mediated ADP (S3 Fig).

### HIV-1-specific antibodies efficiently capture HIV-1_iGFP/JR-FL_ and HIV-1_iGFP/NL4-3_ virions

**Fig 2:**
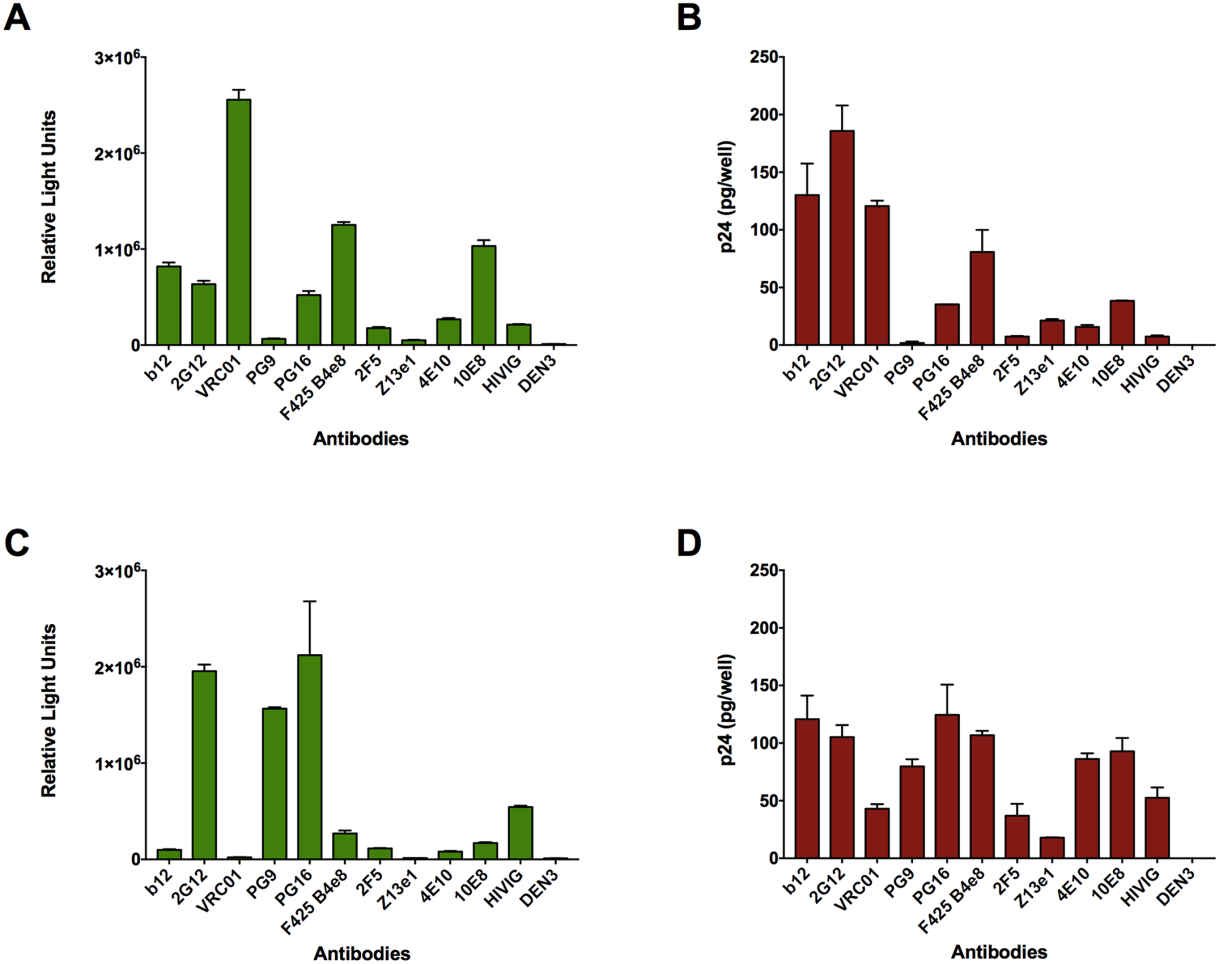
Antibodies variably capture HIV-1 virions. HIV-specific antibodies were tested for their ability to capture infectious HIV-1_iGFP/JR-FL_ by determining the quantity of bound virus that infects TZM-bl cells (**A**). Total virus capture (without regard to infectivity) was measured by determining p24 content of captured virus by ELISA **(B)**. Similarly, capture of infectious (**C**) or total (**D**) HIV-1_iGFP/NL4-3_ was determined. RLU refers to relative light units. All antibodies were tested at a concentration of 0.002 mg/mL. Experiments were performed in triplicate and repeated three times. Data represent means + SEM.

Although unlikely for neutralizing antibodies, the lack of ADP could be explained by insufficient capture of virus. Thus, we examined the antibodies for their ability to capture HIV-1_iGFP/JR-FL_ and HIV-1_iGFP/NL4-3_. For this approach, we coated ELISA plates with an anti-human IgG antibody and stepwise added the HIV-specific antibodies and the GFP-expressing virus. We then either added TZM-bl cells to measure the quantity of infectious virus captured, or we directly assayed bound p24 as a measure of total virus captured by the antibodies. As expected, the antibodies varied in their ability to capture virus (Fig 2). Moreover, the amount of infectious virus captured generally correlated with the amount of total virus captured (*r* = 0.89; *p*<0.0001 for HIV-1_iGFP/JR-FL_ and *r* = 0.75; *p* = 0.002 for HIV-1_iGFP/NL4-3;_ compare Fig 2A to 2B and Fig 2C to 2D). The most notable difference in capture when the two different viruses were compared occurred with the gp120-specific monoclonal antibody PG9, which poorly captured HIV-1_iGFP/JR-FL_ but readily captured HIV-1_iGFP/NL4-3_. Such a difference is consistent with reports of HIV-1_JRFL_ resistance to neutralization by PG9 [22, 28]. These data clearly indicate that most of the antibodies were able to bind to and capture virions. However, despite the capture, the antibodies were incapable of mediating ADP.

### Antibody-dependent phagocytosis relies on an adequate amount of antigen on the viral surface

Based on the fact that most of the antibodies bound to and captured infectious virus yet were unable to mediate ADP, we wondered if the low abundance of envelope glycoproteins (i.e., an average of 7–14 spikes per virion [29]) on the virus surface might play a role in evading ADP. To address this question, we directly coated HIV-1_iGFP/JR-FL_ with a 27-mer peptide mimicking the MPER (LLELDKWASLWNWFDITNWLWYIK-KKK) or with HIV-1_MN_ gp41 protein. By ELISA, all MPER-specific antibodies, including Z13e1, 2F5, 4E10, and 10E8 reacted with the MPER peptide (S4 Fig). In the case of HIV-1_MN_ gp41, we found binding with antibodies Z13e1, 2F5, and 4E10, as well as with the polyclonal antibody HIVIG (S4 Fig). As expected, DEN3 and the gp120-specific antibodies bound poorly to either the MPER peptide or to gp41.

**Fig 3:**
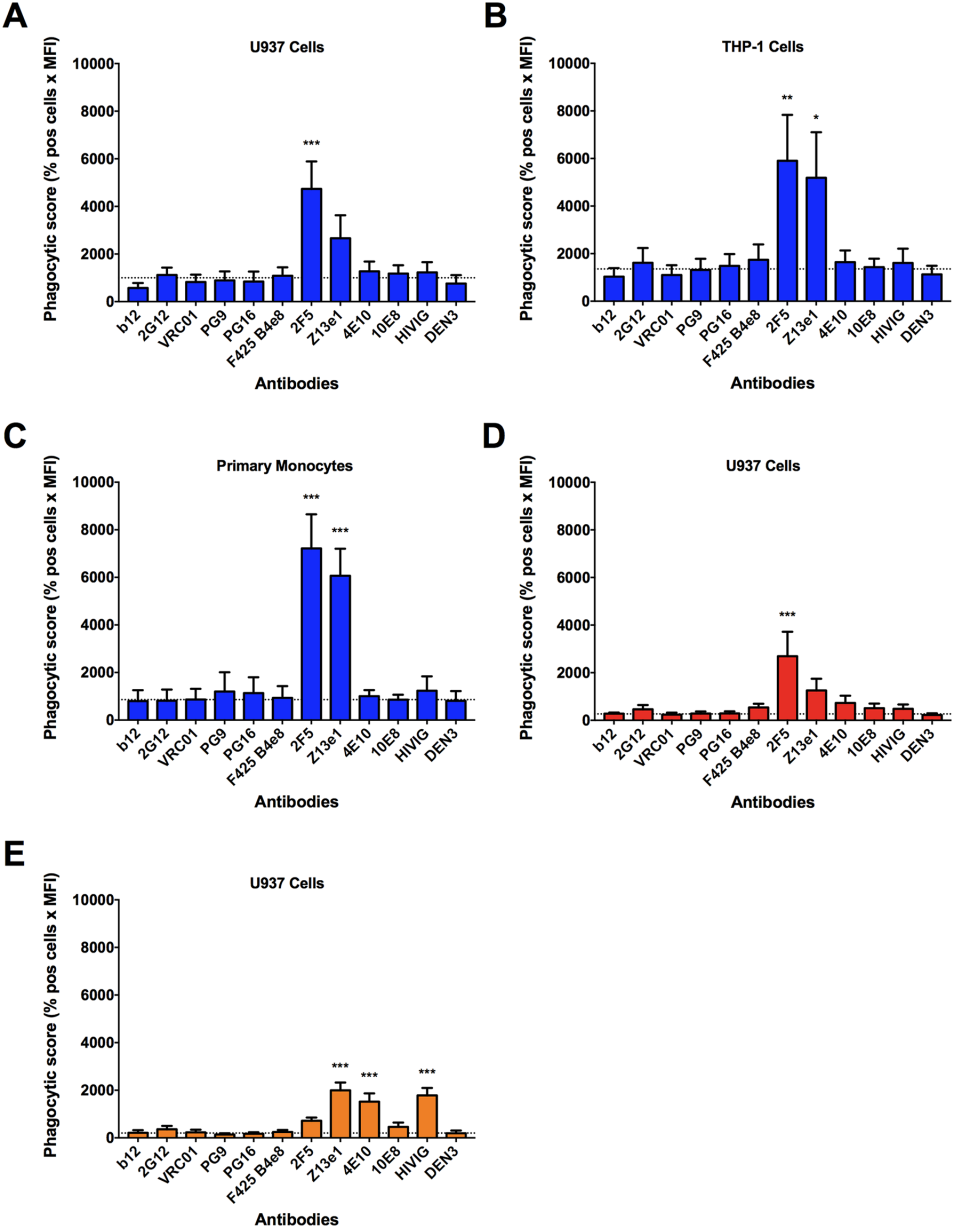
Coating virus with gp41-derived peptide or gp41 results in phagocytosis by gp41-specific antibodies. The gp41-specific mAbs 2F5 and Z13e1 mediate the internalization of MPER peptide-coated HIV-1_iGFP/JR-FL_ by U937 cells **(A)**, THP-1 cells **(B)**, and primary monocytes **(C)**. Similarly, using U937 cells, peptide-coated HIV-1_iGFP/NL4-3_ (**D**) and gp41-coated HIV-1_iGFP/JR-FL_ (**E**) are targets for ADP by gp41-specific mAbs. Antibodies were tested at a concentration of 0.05 mg/mL and the polyclonal antibody HIVIG at a concentration of 0.2 mg/mL. Data is represented as phagocytic score (% positive cells x MFI). One-way ANOVA was used in the analysis of virus uptake in the presence of an antibody compared to the no antibody control (dotted line). *P*-values are indicated with an asterisk: * *p* ≤ 0.05; ** *p* ≤ 0.01, and *** *p* ≤ 0.001. Experiments were performed in triplicate and repeated at least three times. Data represent means + SEM.

Unlike the case with virus alone, HIV-1_iGFP/JR-FL_ coated with MPER peptide was clearly internalized by the gp41-specific antibodies 2F5 and Z13e1; this effect was somewhat less apparent with U937 cells than with either THP-1 cells or primary monocytes (Fig 3A, 3B and 3C). The limited binding of 4E10 and 10E8 to MPER peptide (S4 Fig) likely explains the lack of ADP mediated by those antibodies against the coated virus. Similar results were found using HIV-1_iGFP/NL4-3_ coated with MPER peptide (Fig 3D). As expected, there was no ADP mediated by the gp120-specific antibodies.

To further demonstrate that increasing the amount of antigen on the surface of virions allows ADP to occur, we determined the uptake of virions coated with HIV-1_MN_ gp41. Again, the gp41-specific mAbs, as well as HIVIG, mediated ADP, whereas the gp120-specific mAbs did not (Fig 3E). We also investigated virus internalization before and after coating with gp41 using imaging flow cytometry. We found that a gp41-specific antibody (Z13e1) yielded a higher amount of internalized gp41-coated virions than uncoated virions (S5 Fig). Z13e1 also allowed more internalization of gp41-coated virus than did control antibody (DEN3) or no antibody. These findings further support the notion that a higher density of HIV-1 antigen is required for ADP.

We also used influenza virus, which has about 30-fold more spikes per virion than does HIV-1, to support our finding that antigen density or number is a key factor in successful ADP. Influenza virus uptake was measured by quantitative RT-PCR of lysed THP-1 cells, which avoided the need to label virus (see also above regarding phagocytosis of unlabeled HIV-1 grown in PBMCs). All three anti-hemagglutinin mAbs mediated ADP, resulting in 2 to 3-fold increases in internalization compared to a negative control antibody or to no antibody (S6 Fig).

To assess the approximate number of HIV-1_MN_ gp41 molecules that are required to induce ADP, we analyzed the unbound fraction of gp41 protein left in the supernatant after coating the HIV-1_iGFP/JR-FL_ virions. We found that approximately 540 (±29) gp41 molecules per virion were necessary to elicit the levels of ADP observed in Fig 3E. Note that this number of gp41 molecules does not necessarily represent a minimum value for ADP, since fewer molecules could still result in some degree of antibody-mediated internalization of virus.

### Aggregation of HIV-1 virions significantly enhances antibody-dependent phagocytosis

**Fig 4:**
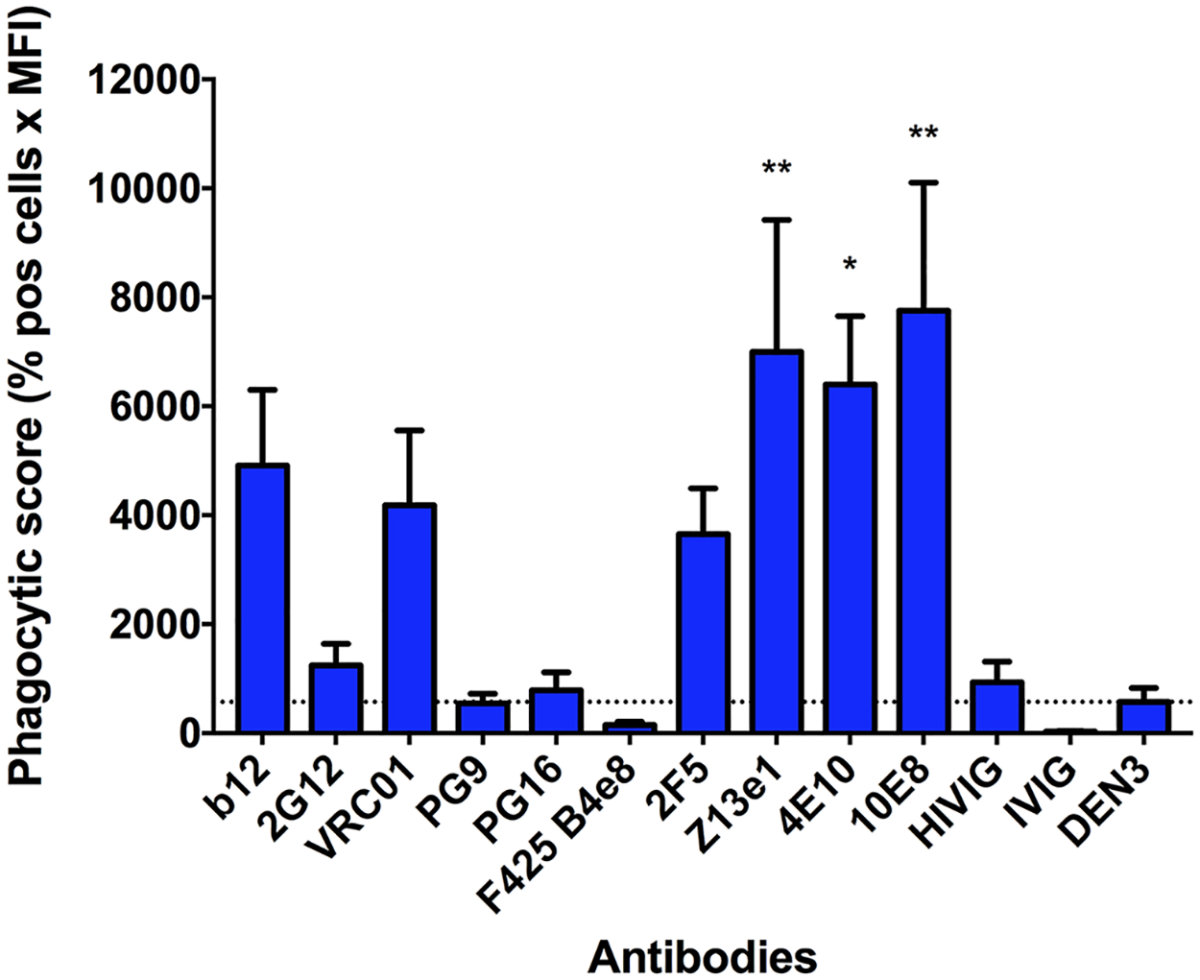
Antibodies mediate phagocytosis of aggregated virus. To induce aggregation, HIV-1_iGFP/JR-FL_ virions were opsonized with HIV-specific antibodies and subsequently incubated with a goat IgG anti-human Fc-specific antibody (0.15 mg/mL) prior to being fed to U937 cells. All mAbs were tested at a concentration of 0.05 mg/mL and the polyclonal antibodies HIVIG and IVIG at a concentration of 0.2 mg/mL. Data are represented as phagocytic score (% positive cells x MFI). One-way ANOVA was used in the analysis of virus uptake in the presence of an antibody compared to the DEN3 control mAb (dotted line; which was higher than the no antibody control). *P*-values are indicated with an asterisk: * *p* ≤ 0.05; ** *p* ≤ 0.01, and *** *p* ≤ 0.001. Experiments were performed in triplicate and repeated at least five times. Data are reported as means + SEM.

The increased antigen on the surface of virions that is required for effective ADP suggests that antibody-mediated internalization is dependent on a threshold number of bound antibodies or on the aggregation of viral particles that requires a threshold number of bound antibodies. To elucidate the role of aggregation in ADP, we first artificially aggregated antibody-opsonized HIV-1 using a goat IgG directed against human Fc. Aggregated antibody-virus complexes were fed to U937 cells, and ADP was measured by flow cytometry as in previous experiments. We found a marked increase in phagocytosis levels for most of the antibodies tested (Fig 4). Consistent with its poor binding to HIV-1_iGFP/JR-FL_, PG9 induced only background levels of phagocytosis similar to the negative control antibody. Interestingly, and for unclear reasons, antibodies PG16, 2G12, and F425 B4e8 also did not mediate ADP of the aggregated complexes above background levels. We also used a goat F(ab’)_2_ directed against human F(ab’)_2_ in an attempt to aggregate opsonized virus. This resulted again in ADP, albeit to a substantially lower degree compared with the goat IgG (S7 Fig). We did not directly measure aggregation using the F(ab’)_2_, and the lower level of ADP may be explained by: 1) the goat IgG (rather than the human anti-HIV antibodies) is primarily responsible for interacting with the FcγRs on the phagocytes; 2) the goat IgG is better than the F(ab’)_2_s at aggregating virus; 3) the goat IgG allows a better orientation of the human antibodies for engaging the FcγRs; or 4) some combination of the above. In any case, these results overall indicate that virus aggregation can result in ADP in the presence of HIV-1-specifc antibodies. However, whether sufficient aggregation might occur in nature is unknown. In this regard, it is of interest that pools of hundreds of HIV-1 virions can be seen in gut-associated lymphoid tissue and spleens of HIV-infected humanized mice [30, 31].

**Fig 5:**
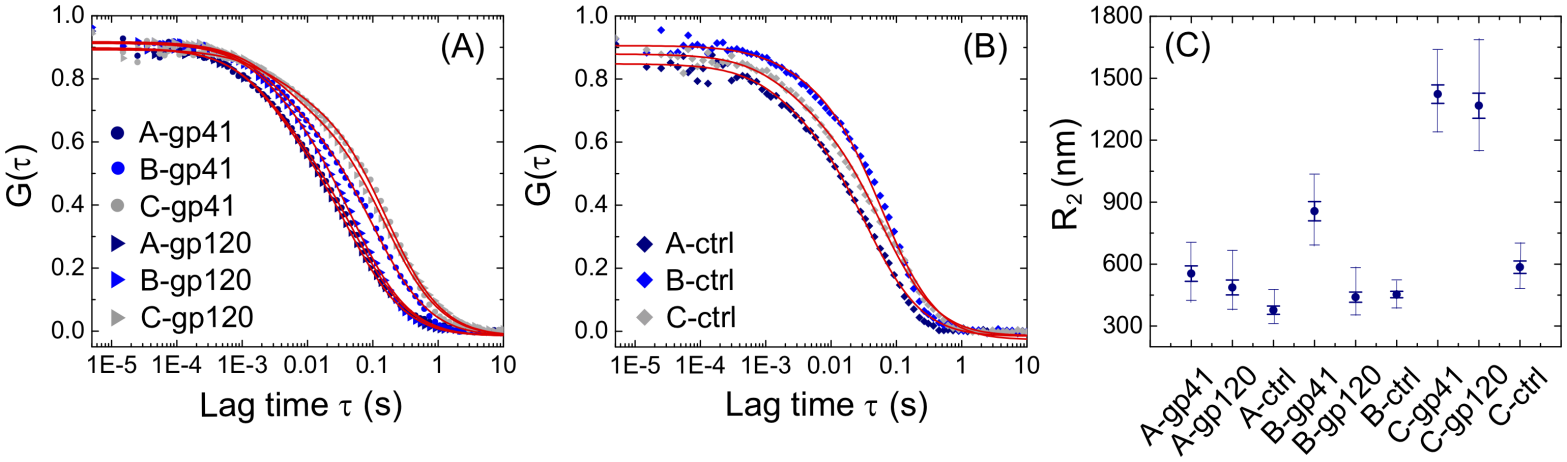
Fluorescence correlation spectroscopy reveals quantitative differences in virion aggregation. (**A**) Normalized experimental average ACFs recovered for native virus opsonized with anti-gp41 mAbs or HIVIG (A-gp41) or with anti-gp120 mAbs or HIVIG (A-gp120), gp41-coated virus opsonized with anti-gp41 mAbs or HIVIG (B-gp41) or with anti-gp120 mAbs or HIVIG (B-gp120), aggregated virus opsonized with anti-gp41 mAbs or HIVIG (C-gp41) or with anti-gp120 mAbs or HIVIG (C-gp120). (**B**) Normalized experimental average ACFs recovered for control groups consisting of the same virus preparations as in (**A**) but with no antibody or anti-dengue mAb. In both panels (**A**) and (**B**), only half of the total data points is shown for the sake of visual clarity, and the ACFs global fit to eq. S.4 (S1 Text) is shown with red continuous lines (all best-fit parameters are reported in S1 Table). (**C**) Hydrodynamic radius R_2_ of the aggregate population recovered for each group. Radii were computed based on the Stokes-Einstein equation (S1 Text) from the best-fit estimates of the diffusion coefficient D_2_. Thick error bars correspond to the standard deviations quantified by the rigorous error analysis of the ACF global fit as described in Materials and Methods. The same rigorous error analysis has also been exploited to yield, for each hydrodynamic radius, the minimum and maximum allowed values (thin error bars) compatible with one standard deviation of the ACF’s global-fit chi-square value. Results of the one-way ANOVA statistical analysis on the recovered radii are reported in S2 Table. All FCS experiments were conducted twice.

In order to clarify whether aggregation plays a role in the antibody-mediated phagocytosis of the MPER- or gp41-coated virions, we applied fluorescence correlation spectroscopy (FCS), a well-established technique to measure average particle size and monitor aggregation, to our virus preparations [23, 24, 32]. We analyzed three groups of antibody-opsonized virions: A) native (i.e., uncoated) virus opsonized with the panel of mAbs or HIVIG; B) virus coated with gp41 and opsonized with the antibodies; and C) native virus opsonized with the antibodies and aggregated with an anti-human Fc antibody. Negative controls consisted of non-opsonized virions (i.e., without antibody or with the anti-dengue virus antibody DEN3). For all the samples in each group, auto correlation functions (ACFs) were normalized and averaged to yield a single ACF (Fig 5A and 5B; S8 Fig). A global fit of the nine ACFs to a double-component diffusive model (eq. S.4 in S1 Text; S2 Fig) was performed to yield the average diffusion coefficient and hydrodynamic radius of the two diffusing populations (Fig 5A and 5B). While we report all the best-fit parameters (S1 Table), we focus here on the aggregates’ hydrodynamic radius (R_2_) (Fig 5C). Our results show that R_2_ for group B (gp41-coated virus) opsonized with anti-gp41 antibody was significantly larger than R_2_ for group A (native virus) opsonized with anti-gp41 antibody (R_2_ = 863 ± 47 nm and 558 ± 37 nm for groups B and A, respectively; p <0.0001; Fig 5C; S2 Table). Thus, the addition of anti-gp41 antibody to gp41-coated virions promotes aggregation and, as shown above, allows ADP (Fig 3). By contrast, there was no significant difference between group A and group B virus opsonized with anti-gp120 antibody, and unopsonized group B virus gave similar results as anti-gp120-opsonized group B virus (Fig 5C; S2 Table). Strong aggregation was observed for group C virus whether opsonized with anti-gp41 or anti-gp120 antibodies (Fig 5C and S2 Table), consistent with the likely ability of anti-human antibody to crosslink Env-specific antibodies bound to different virions. Notably, phagocytosis of opsonized group C virus was more efficient than for other conditions (Fig 4). Taken together, these observations indicate that conditions resulting in the aggregation of virions also result in ADP.

### Opsonized, HIV-1-coated cells are targets for ADP or trogocytosis

**Fig 6:**
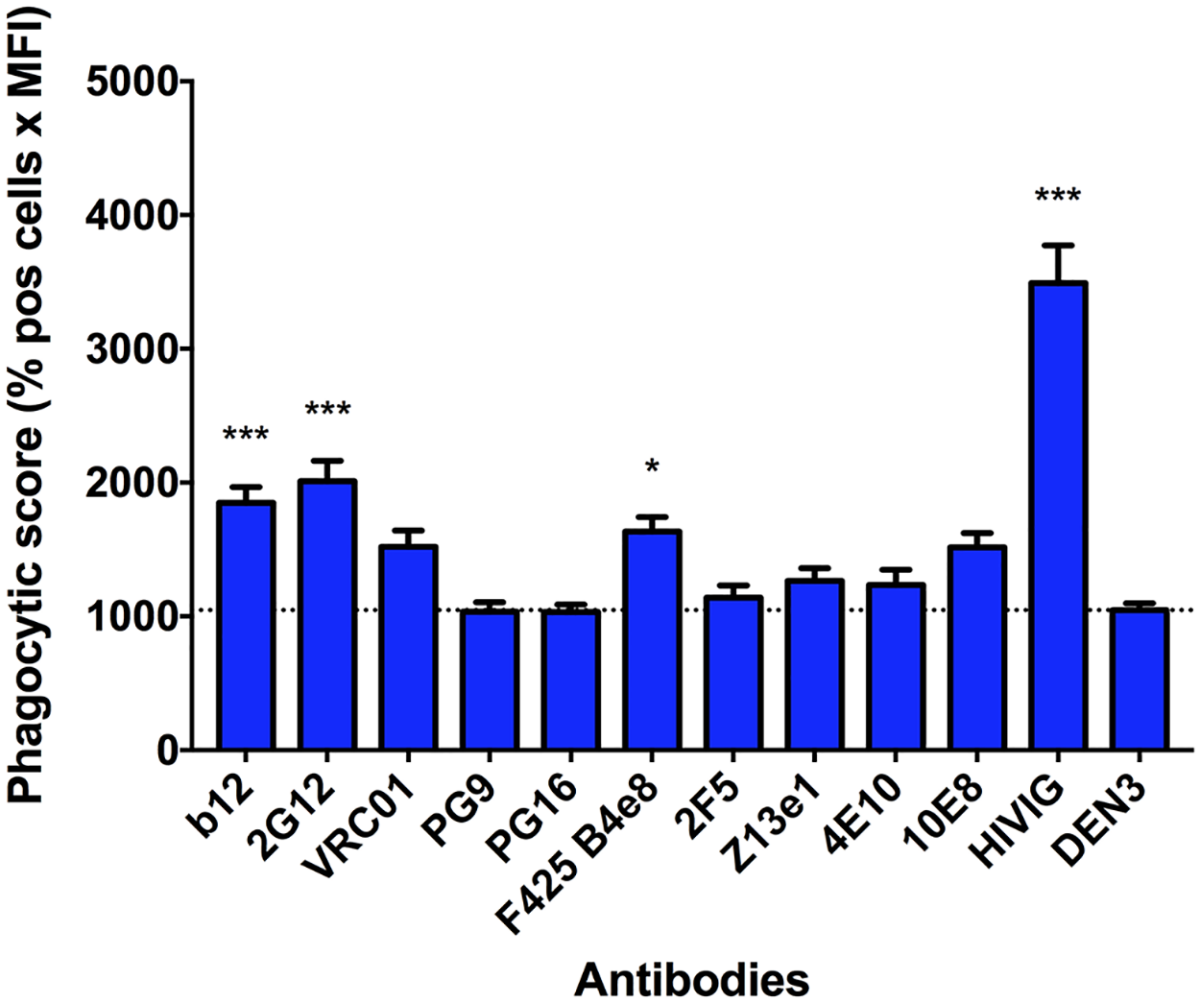
CEM.NKr-CCR5 cells decorated with HIV-1_iGFP/JR-FL_ are targets for ADP or trogocytosis by Env-specific antibodies. HIV-1_iGFP/JR-FL_ virions were spinoculated onto CEM.NKr-CCR5 target cells at 4°C, which were then opsonized with HIV-specific antibodies. THP-1 effector cells (effector:target ratio = 1:1) were next incubated with the target cells for one hour. The THP-1 cells were identified by PE-conjugated anti-CD32 staining, and double positive (PE+/GFP+) cells were analyzed using flow cytometry. Antibodies were tested at a concentration of 0.05 mg/mL and the polyclonal antibody HIVIG at a concentration of 0.2 mg/mL. Data are represented as phagocytic score (% positive cells x MFI). Note that these methods do not distinguish whether target cells themselves are internalized (phagocytosis) or whether membranes containing virus are internalized (trogocytosis). One-way ANOVA was used to analyze differences compared with the no antibody control (dotted line), after demonstrating a normal data distribution. *P*-values are indicated with an asterisk: * *p* ≤ 0.05; ** *p* ≤ 0.01, and *** *p* ≤ 0.001. Experiments were performed in triplicates and repeated at least four times. Data are means + S.E.M.

We next investigated whether CEM.NKr-CCR5 cells coated with HIV-1_iGFP/JR-FL_ at 4°C (so as to inhibit entry) would serve as targets of antibody-dependent internalization. This set of experiments was based on the notion that antibodies might still provide a mechanism of clearing virus prior to establishing infection. Thus, in the absence of antibody-mediated clearance of virions, phagocytosis or trogocytosis of cells in the process of becoming infected could be involved in the observed importance of Fc-FcγR interactions in augmenting the anti-viral effect of neutralizing antibodies *in vivo* [33, 34]. Antibody-opsonized, HIV-1_iGFP/JR-FL_-coated CEM.NKr-CCR5 cells were incubated with THP-1 cells, and flow cytometry was used to quantify internalized iGFP signal by the phagocytic THP-1 cells (stained with PE-conjugated anti-CD32). We found a broad range of internalization mediated by the different antibodies in our panel, with b12, 2G12, VRC01, and HIVIG performing the best (Fig 6; S9 Fig). We note that our methods do not distinguish internalization of the virus-decorated CEM.NKr-CCR5 target cells themselves from trogocytosis of the target-cell surface membrane that contains iGFP-expressing virions.

### Methods relying on spinoculation or on membrane dye- or FITC-labeling of virus may bias ADP results

To determine if virions of clade B HIV-1 strains other than HIV-1_JRFL_ and HIV-1_NL4-3_, which were not available as iGFP-expressing virus, were also poor targets for phagocytosis, we labeled infectious molecular clones HIV-1_HXB2_, HIV-1_SF162_, HIV-1_JR-CSF_, and HIV-1_ADA_ with a fluorescent membrane dye (DiD) and measured the cellular uptake of labeled virions in the presence and absence of HIV-1-specific antibodies. Under these conditions, PG9- and 10E8-opsonized HIV-1 was internalized very efficiently by THP-1 cells, whereas the other gp120-specific antibodies resulted in poor internalization (S10 Fig). Surprised by these results, we became concerned that the antibodies might be reacting with the DiD membrane dye itself. To test this possibility, we generated pseudotyped virus composed of the HIV-1 core (no HIV-1 envelope) and the envelope glycoprotein of vesicular stomatitis virus (VSV-G). After labeling with DiD, we found very effective uptake of PG9- and 10E8-opsonized, VSV-G-enveloped pseudotyped virus (S10 Fig). These results indicate that at least some antibodies will non-specifically interact with DiD, arguing against the use of that membrane dye for ADP experiments. We did not investigate further the mechanisms of this non-specific interaction.

We and others have used FITC to label virus for ADP studies, and concern about the membrane dye led us to evaluate this method further. Using FCS again, we found that FITC labeling resulted in substantial aggregation of virus independently of antibody opsonization (S11 Fig). Since aggregated virions are readily internalized (Fig 4), and there is no reason to suspect that this type of aggregation occurs in nature, the use of FITC-labeled virus is likely to give false-positive results in phagocytosis assays.

In contrast to our results, a recent study found that ADP does, in fact, occur with HIV-1 virions [19]. That study measured phagocytosis of HIV-1 virus after spinning antibody-opsonized and unopsonized virus onto THP-1 cells, a procedure that we did not employ. However, we found that the same spinning step had a differential effect on antibody-opsonized virus compared with unopsonized virus, allowing substantially more of the opsonized virus (reported as the phagocytic score) to stick to the surface of THP-1 cells prior to phagocytosis (S12 Fig). Thus, spinning of virus particles may bias phagocytosis assays toward a positive ADP result, simply by allowing more opsonized than unopsonized virus to have contact with the phagocytes.

## Discussion

Antibody-dependent phagocytosis is thought to contribute to the *in vivo* control of viral infections such as those caused by influenza and West Nile viruses [13, 35, 36]. In contrast, the role of phagocytosis in clearance of HIV-1 remains unknown. In this study, we sought to directly measure *in vitro* the ability of antibodies to mediate the internalization of HIV-1 by phagocytic cells. Our results indicate that antibody opsonization does not, in fact, increase the uptake of virus particles when compared to unopsonized virus. Minimal or absent ADP was demonstrated for several antibodies capable of binding to and capturing infectious virus. Furthermore, ADP was negligible using two different phagocytic cell lines and primary monocytes. Successful ADP following the coating of virions with gp41 or a gp41-derived peptide suggested that the paucity of Env spikes on native virions was a major contributor to the observed lack of phagocytosis. In contrast to free virions, internalization of virus was observed using T cells adsorbed with HIV-1, likely by phagocytosis of the T cell or by trogocytosis of T cell membranes containing virus.

Our observation that antibody-opsonized HIV-1 is a poor target for phagocytosis is in contrast to what other studies have demonstrated. In such a study by Tay et al. using mCherry-labeled HIV-1 strains different than those employed in our study, ADP was demonstrated for a number of mAbs [19]. However, in that study, opsonized virus and unopsonized virus used as a control were spun with THP-1 effector cells at 1200 g for 1 hour. We have shown that such a spin results in a greater quantity of opsonized virus alighting on the effector cells than unopsonized virus (S12 Fig). The greater amount of virus on the effector-cell surface would provide a greater opportunity for internalization of the opsonized virus even if the internalization is mediated by endocytic signals unrelated to antibody and FcγRs. Thus, spinning very likely results in a bias toward greater internalization of immune complexes. Our data also suggest that the use of FITC-labeled virus may not be appropriate for measuring ADP. FITC itself results in aggregation of HIV-1 virions (S11 Fig), and aggregated virus is readily phagocytosed (Fig 4); we did not determine if FITC labeling also aggregates influenza virus, where FITC has also been used to demonstrate ADP, though this seems likely given previous reports of protein crosslinking in general by FITC [37]. Finally, we attempted to use a membrane dye in an effort to extend ADP assays to any strain of HIV-1 without the requirement for engineering. However, again, such labeling introduced an artifact that led to the false impression that certain antibodies could mediate ADP (S10 Fig).

The poor phagocytosis of opsonized HIV-1 virions cannot be attributed to poor capture by the antibodies we used, since several of them, as expected by their neutralizing activity, were quite efficient at capture. Rather, the paucity of Env spikes offers a likely explanation. The direct coating of virions with gp41 or gp41-derived peptide allowed ADP by gp41-specific antibodies, suggesting that a threshold level of antigen on the virion surface is required. Our results with fluorescence correlation spectroscopy showed a difference in aggregation between antibody-opsonized and unopsonized virus that had been coated with gp41. On the other hand, opsonized virus that is not coated with extra antigen (i.e., native virus) does not form significantly more aggregates than does unopsonized, native virus. Thus, additional antigen is required for ADP and additional antigen is also required for aggregation. Our methods cannot distinguish whether non-aggregated, antigen-coated virus is also a target for ADP, and it is possible that the addition of antigen by itself (independently of aggregation) provides the appropriate orientation or spacing of Fc with respect to FcγRs to allow phagocytosis.

By providing a means of evading phagocytosis, the paucity of Env spikes appears to be a way in which HIV-1 circumvents its destruction by phagocytes. HIV-1 transmission may occur after the development of an antibody response, and it is likely, though not proven, that virus in mucosal secretions is often in the form of an immune complex. If antibody-bound virus were readily phagocytosed, transmission might be less effective than it is known to be. Although it is possible that phagocytosis itself could enhance transmission, our findings indicate that evasion of phagocytosis might be a feature of HIV-1’s success as a sexually and mother-to-child-transmitted organism.

Although our results suggest that ADP might not be an important means of clearing HIV-1, a study by Bournazos, et al. [34] using a mouse model found that mAbs do participate in the clearance of virions in an FcγR-dependent manner. However, that study was conducted using pseudotyped virus, which may have a substantially higher density of Env spikes than authentic virus [38]. Furthermore, it is possible that the observed viral clearance occurred either by trogocytosis or because of the elimination of cells that were bound with virus (by ADCC [39, 40] or by phagocytosis). In this regard, our results demonstrate that, unlike free virions, cells with surface-attached virions provide a target for antibody-dependent internalization of virus, either by trogocytosis of T cell membranes containing virions or by phagocytosis of the T cells themselves. In either case, it would appear that adsorbing virus onto T cells aggregates virions or otherwise allows bound antibody to crosslink FcγRs.

Although we found no virion ADP using two phagocytic cell lines or primary monocytes, we do not leave out the possibility that other effector cells could mediate ADP or that *in vivo* conditions allow aggregation or some other means of clearance of virions through ADP. Nonetheless, taken together, our findings suggest that FcγR-dependent functions other than ADP of free virions may be critical for maximal prevention or control of HIV-1 infection by antibody.

## Supporting information

S1 Fig Representative flow cytometry plots illustrating gating strategies. (**A**) For HIV-1_iGFP_ phagocytosis, cells were gated based on FSC/SSC profile, and shifts in the FITC-GFP channel (virus internalization) were measured (left panels). Representative histograms are shown (right). (**B**) For phagocytosis of cells decorated with HIV-1, singlet events were gated based on FSC/SSC profiles to discriminate cells from debris. THP-1 cells were then gated on the basis of positive anti-CD32 PE staining, and PE-positive cells were evaluated for GFP signal (internalization of virus-decorated cells or fragments of cells).
(PDF)

S2 Fig Single-component diffusive model versus double-component diffusive model for FCS non-linear curve fitting. (**A**) The normalized average ACF for 2F5-, Z13e1-, 4E10-, 10E8- and HIVIG-opsonized native virions (group A) is shown (navy circles, mean ± SEM) with a fit to the one-component free three-dimensional Brownian diffusion model (equation in S1 Text; red dashed-dotted line; best-fit parameters are G0 = 0.940±0.007 and D = 1.00 ± 0.03 μm^2^/s). A satisfactory fit of experimental data and uncorrelated fit residuals can only be achieved by employing a two-component free three-dimensional Brownian diffusion model (red continuous line; best-fit parameters are reported in S1 Table). As shown here for group A virus opsonized with anti-gp41 antibody, it is necessary to employ a two-component fitting model to all the virion preparations. An example of a normalized ACF (pink triangles) measured on reference 100-nm yellow-green fluorescent beads (mean ± SEM, total sampling time 520 s) is shown overlaid to fit eq. S. 2 (S1 Text) (red continuous line; best fit parameters are G0 = 0.987 ± 0.002 and ω_0_ = 0.311 ± 0.001 μm; D = 4.34 μm^2^/s was treated as fixed fit parameter for the beam waist calibration). (**B**) Example of raw confocal image acquired on antibody-opsonized virions cast on a glass coverslip. The setup consisted of a Zeiss 880 laser scanning confocal microscope with excitation wavelength = 488 nm; pinhole size = 1 Airy Unit, producing a spatial resolution of ~200 nm; image format = 1024 x 1024; pixel dwell time = 2 μs; 40x water immersion objective lens. (**C**,**D**) Fluorescence intensity profiles (black) extracted across two imaged particles (intensity profiles were extracted along the two yellow lines shown in panel **B**), overlaid to their fit to a Gaussian function (red). The distance from the profile peak (i.e., the center of the imaged particle) to the point where the intensity drops to 1/e2 = 13.7% provides a possible estimate for the particle radius and equals 435 ± 1 nm and 393 ± 1 nm in panels **C** and **D**, respectively. A single opsonized virion cannot produce such an intensity profile. Since antibodies are not fluorescent, the fluorescence signal only arises from the virion itself, which is smaller than the 200 nm diffraction-limited spatial resolution of the employed confocal microscope. Irrespectively of how many antibodies surround the virion and how big the entire complex is, the whole object still acts as a sub-resolved particle for the confocal microscope. It cannot appear bigger than 200 nm. Only aggregates made up of multiple fluorescent virions can appear as spots larger than 200 nm in confocal images.
(PDF)

S3 Fig HIV-1 envelope-specific antibodies do not mediate phagocytosis of PBMC-grown virus. Uptake of HIV-1_JRFl_ virions was measured in U937 cells by quantitative RT-PCR. All mAbs were tested at a concentration of 0.02 mg/mL, and the polyclonal antibodies HIVIG and IVIG at a concentration of 0.08 mg/mL. PGN635 was used as a positive control. Data are reported as viral RNA copy numbers per 25,000 cells. One-way ANOVA was used to analyze virus uptake in the presence of antibody compared to the no antibody control (dotted line). *P*-values are indicated with an asterisk: * *p* ≤ 0.05; ** *p* ≤ 0.01, and *** *p* ≤ 0.001. All phagocytosis experiments were performed in triplicate and repeated at least three times. Data are reported as means + SEM.
(PDF)

S4 Fig gp41-specific mAbs and HIVIG bind to gp41 and the gp41-derived peptide used to coat virions. MPER peptide captured with NeutrAvidin (**A**) or recombinant HIV-1_MN_ gp41 protein captured with D50 antibody (**B**) was used to measure antibody binding by ELISA. Data represent the means from two independent experiments in duplicate.
(PDF)

S5 Fig Opsonized, gp41-coated virions are internalized by THP-1 cells. Image Stream was used to quantify Z13e1-opsonized or unopsonized virus internalized by THP-1 effector cells. Internalization of positive events was measured by applying the ImageStream IDEAS Internalization and Spot Wizard algorithms, which defined the internal area as a mask of erosion of 4 pixels into the brightfield perimeter of the cell. According to the protocol outlined in [41], an internalization score of 0.3 and a spot count of 3 were used to identify internalized virions and exclude surface-bound virions and background fluorescence. The gating strategy (**A**), a representative image (**B**), and percent of cells with internalized virus compared to the total number of focused, single cells (**C**) are shown. More than 10,000 images per condition were collected.
(PDF)

S6 Fig mAbs directed against influenza virus hemagglutinin mediate ADP of influenza virus. Internalization of influenza virus was measured by qPCR of lysed THP-1 cells following a 60- to 80-minute period of exposure of cells to virus opsonized with anti-hemagglutinin mAbs FI6, 2G02, and CR9114. All mAbs were tested at a concentration of 0.01 mg/mL. Data represent means + SEM of duplicate independent experiments, each performed in triplicate.
(PDF)

S7 Fig Antibodies mediate phagocytosis of aggregated HIV-1 virions. To induce aggregation, HIV-1_iGFP/JR-FL_ virions were opsonized with HIV-specific antibodies and subsequently incubated with a goat F(ab’)_2_ anti-human F(ab’)_2_ antibody (0.05 mg/mL) prior to being fed to U937 cells. All mAbs were tested at a concentration of 0.05 mg/mL and the polyclonal antibodies HIVIG and IVIG at a concentration of 0.2 mg/mL. One-way ANOVA was used to analyze virus uptake in the presence of antibody compared to the DEN3 control mAb (dotted line). *P*-values are indicated with an asterisk: * *p* ≤ 0.05; ** *p* ≤ 0.01, and *** *p* ≤ 0.001. Experiments were performed in triplicate and repeated at least twice. Data are reported as means + SEM.
(PDF)

S8 Fig FCS correlation function standard error. Normalized experimental average ACFs recovered for group A, B, and C virus opsonized with anti-gp41 antibody (**A**), anti-gp120 antibody (**B**) or unopsonized (**C**). Data are reported as mean ± SEM. Only half of the correlation data points are reported for the sake of visual clarity.
(PDF)

S9 Fig CEM.NKr-CCR5 cells decorated with HIV-1_iGFP/JR-FL_ are targets for ADP or trogocytosis by Env-specific antibodies. CEM.NKr-CCR5 decorated with virus at 4°C and opsonized with 2G12 or control antibodies were incubated with THP-1 effector cells. THP-1 cells were identified by PE-conjugated anti-CD32 antibody staining. Using ImageStream cytometry, internalization of positive events was measured as described in S5 Fig. The gating strategy (**A**), a representative image (**B**), and the percent of cells with internalized virus compared to the total number of focused, single cells (**C**) are shown.
(PDF)

S10 Fig Membrane dye-labeled HIV-1 phagocytosis. Prior to measuring phagocytosis by THP-1 cells, HIV-1 molecular clones or pseudotyped HIV-1_VSV-g_ (control) were stained with DiD for 30 min at 37°C and subsequently washed with medium. Flow cytometry revealed high levels of ADP in the case of PG9 and 10E8 for all HIV-1 isolates including HIV-1_SF162_ (**A**), HIV-1_HxB2_ (**B**), HIV-1_JR-CSF_ (**C**), and HIV-1_ADA_ (**D**), as well as for the HIV-1_VSV-g_ pseudotyped negative control (**E**). Antibodies were tested at a concentration of 0.05 mg/mL and the polyclonal antibody HIVIG at a concentration of 0.2 mg/mL. The phagocytic score of the no antibody control is indicated by the dotted line. Experiments were performed in triplicate and were repeated at least twice with similar results.
(TIFF)

S11 Fig FITC labeling of virus results in aggregation. Normalized experimental average ACF recovered for FITC-labeled virions freely diffusing in 2% PAF solution is shown. The fit (red continuous line) was performed to eq. S. 4 (S1 Text) with best-fit parameters D_1_ = 1.5 ± 0.5 μm^2^/s, D_2_ = 0.029 ± 0.003 μm^2^/s, G0_1_ = 0.17 ± 0.02 and G0_2_ = 0.72 ± 0.02 μm^2^/s. The corresponding hydrodynamic radii were computed based on the Stokes-Einstein equation as R_1_ = 140±47 nm and R_2_ = 7262±751 nm. Normalized experimental average ACFs measured for opsonized or unopsonized group A, B, and C virus, together with their global fit to eq. S. 4 (S1 Text), are also reported for comparison. Only half of the correlation data points are shown for the sake of visual clarity.
(PDF)

S12 Fig Virus spinoculation onto THP-1 cells results in greater binding of opsonized virus than unopsonized virus. HIV-1_iGFP/JR-FL_ at 10 ng (**A** and **B**) or 50 ng (**C** and **D**) of p24 was opsonized with b12, 2G12, or VRC01 or with DEN3 (unopsonized). Virus-mAb preparations were then either spun in a microfuge at 1200 g for 1 hour with THP-1 cells at 4°C to avoid internalization (**A** and **C**) or left unspun as in all previous experiments (**B** and **D**). Cells were then fixed on ice, and flow cytometry was used to quantify THP-1 cells with virus on their surface. Shown are the means + SEM of five independent experiments. When spinning was employed, each HIV-specific mAb resulted in significantly greater surface binding of HIV-1_iGFP/JR-FL_ than did the DEN3 control mAb (*p* < 0.05). In some cases, there were significant differences between opsonized and unopsonized virus in the absence of spinning (b12 and VRC01 at 10 ng of p24 and 2G12 at 50 ng of p24), whereas there were no significant differences (*p* > 0.05) with 2G12 at 10 ng or with b12 and VRC01 at 50 ng of p24.
(PDF)

S1 Table Best-fit parameters (central column) recovered by the global fit of the average ACFs of groups A, B, or C virus (opsonized with anti-gp41, anti-gp120 antibody, or unopsonized as indicated; ACFs are reported in Fig 5 and S8 Fig). The diffusion coefficient D_1_ of the non-aggregate population and the correlation offset G_∞_ were treated as shared parameters for the global fit of the nine ACFs. Values reported as minimum and maximum (left and right columns, respectively) were obtained by the FCS rigorous error analysis (i.e., they were selected by evaluating all the possible parameter combinations compatible with a predefined maximum variation [one standard deviation] of the ACFs global-fit chi-square value).
(DOCX)

S2 Table One-way ANOVA followed by Tukey’s multiple-comparisons test was performed on the hydrodynamic radii of the aggregate population recovered for all combinations of virus groups and antibody opsonization groups. Radii are reported in Fig 5C). * *p* ≤ 0.0332; ** *p* ≤ 0.0021, *** *p* ≤ 0.0002 and **** *p* ≤ 0.0001).
(DOCX)

S1 Text Fluorescence correlation spectroscopy theory. (DOCX)
