# Supplementary figures and images for: Human immunodeficiency virus type-1 (HIV-1) evades antibody-dependent phagocytosis
Source: PLoS Pathog. 2017 Dec 27;13(12):e1006793. doi: 10.1371/journal.ppat.1006793 (PMC5760106; doi:10.1371/journal.ppat.1006793)

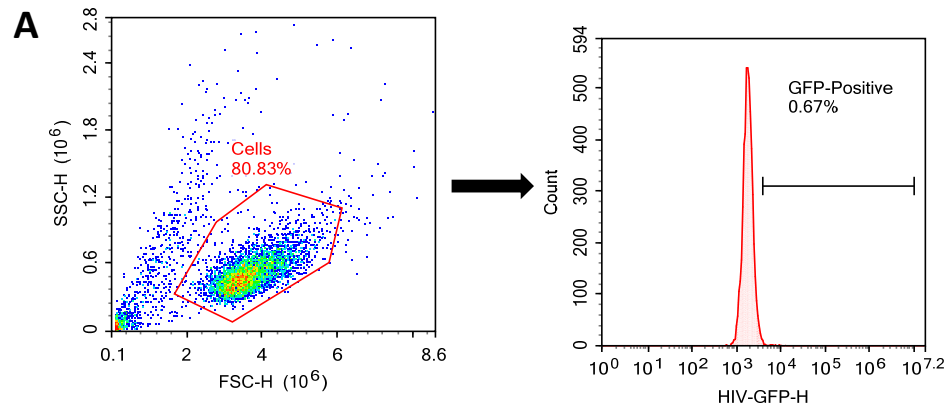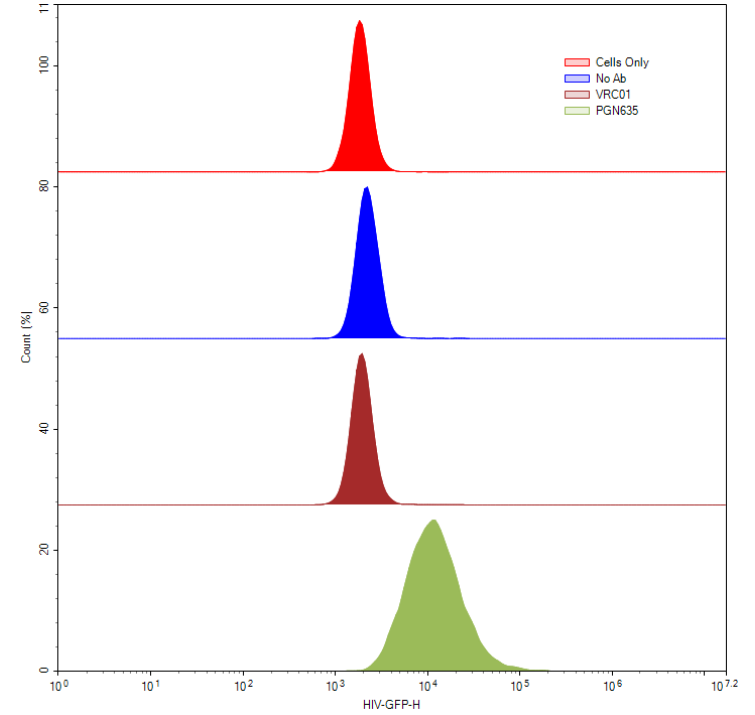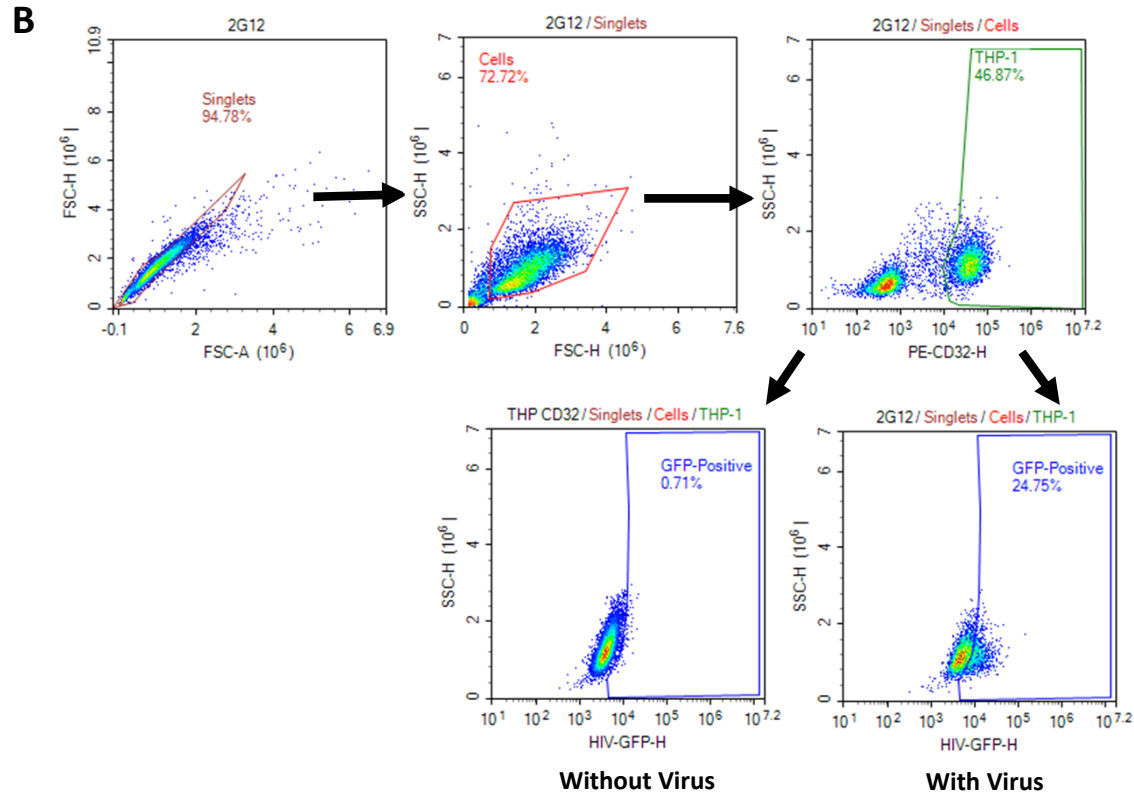

Supplement: S1 Fig — (A) For HIV-1iGFP phagocytosis, cells were gated based on FSC/SSC profile, and shifts in the FITC-GFP channel (virus internalization) were measured (left panels). Representative histograms are shown (right). (B) For phagocytosis of cells decorated with HIV-1, singlet events were gated based on FSC/SSC profiles to discriminate cells from debris. THP-1 cells were then gated on the basis of positive anti-CD32 PE staining, and PE-positive cells were evaluated for GFP signal (internalization of virus-decorated cells or fragments of cells). (PDF) [file ppat.1006793.s001.pdf]

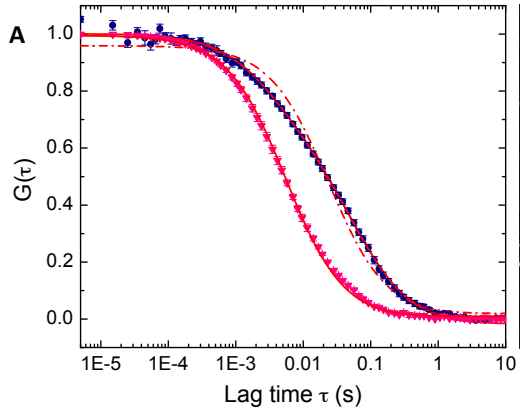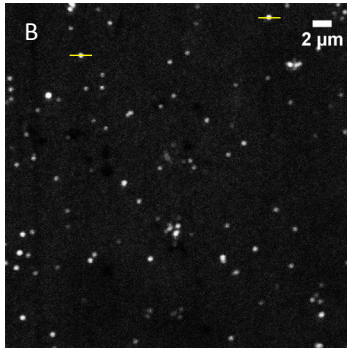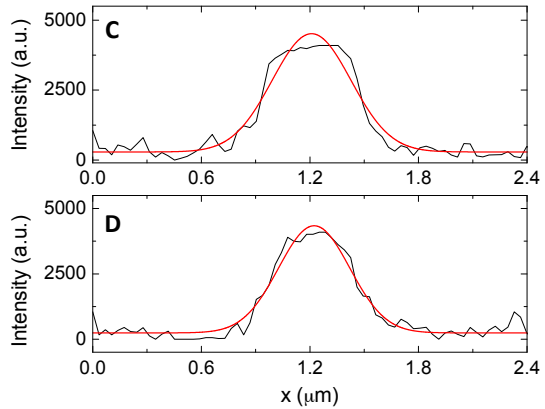

Supplement: S2 Fig — (A) The normalized average ACF for 2F5-, Z13e1-, 4E10-, 10E8- and HIVIG-opsonized native virions (group A) is shown (navy circles, mean ± SEM) with a fit to the one-component free three-dimensional Brownian diffusion model (equation in S1 Text; red dashed-dotted line; best-fit parameters are G0 = 0.940±0.007 and D = 1.00 ± 0.03 μm2/s). A satisfactory fit of experimental data and uncorrelated fit residuals can only be achieved by employing a two-component free three-dimensional Brownian diffusion model (red continuous line; best-fit parameters are reported in S1 Table). As shown here for group A virus opsonized with anti-gp41 antibody, it is necessary to employ a two-component fitting model to all the virion preparations. An example of a normalized ACF (pink triangles) measured on reference 100-nm yellow-green fluorescent beads (mean ± SEM, total sampling time 520 s) is shown overlaid to fit eq. S. 2 (S1 Text) (red continuous line; best fit parameters are G0 = 0.987 ± 0.002 and ω0 = 0.311 ± 0.001 μm; D = 4.34 μm2/s was treated as fixed fit parameter for the beam waist calibration). (B) Example of raw confocal image acquired on antibody-opsonized virions cast on a glass coverslip. The setup consisted of a Zeiss 880 laser scanning confocal microscope with excitation wavelength = 488 nm; pinhole size = 1 Airy Unit, producing a spatial resolution of ~200 nm; image format = 1024 x 1024; pixel dwell time = 2 μs; 40x water immersion objective lens. (C,D) Fluorescence intensity profiles (black) extracted across two imaged particles (intensity profiles were extracted along the two yellow lines shown in panel B), overlaid to their fit to a Gaussian function (red). The distance from the profile peak (i.e., the center of the imaged particle) to the point where the intensity drops to 1/e2 = 13.7% provides a possible estimate for the particle radius and equals 435 ± 1 nm and 393 ± 1 nm in panels C and D, respectively. A single opsonized virion cannot produce such an intensity prof [file ppat.1006793.s002.pdf]

**HIV-1 RNA copies**

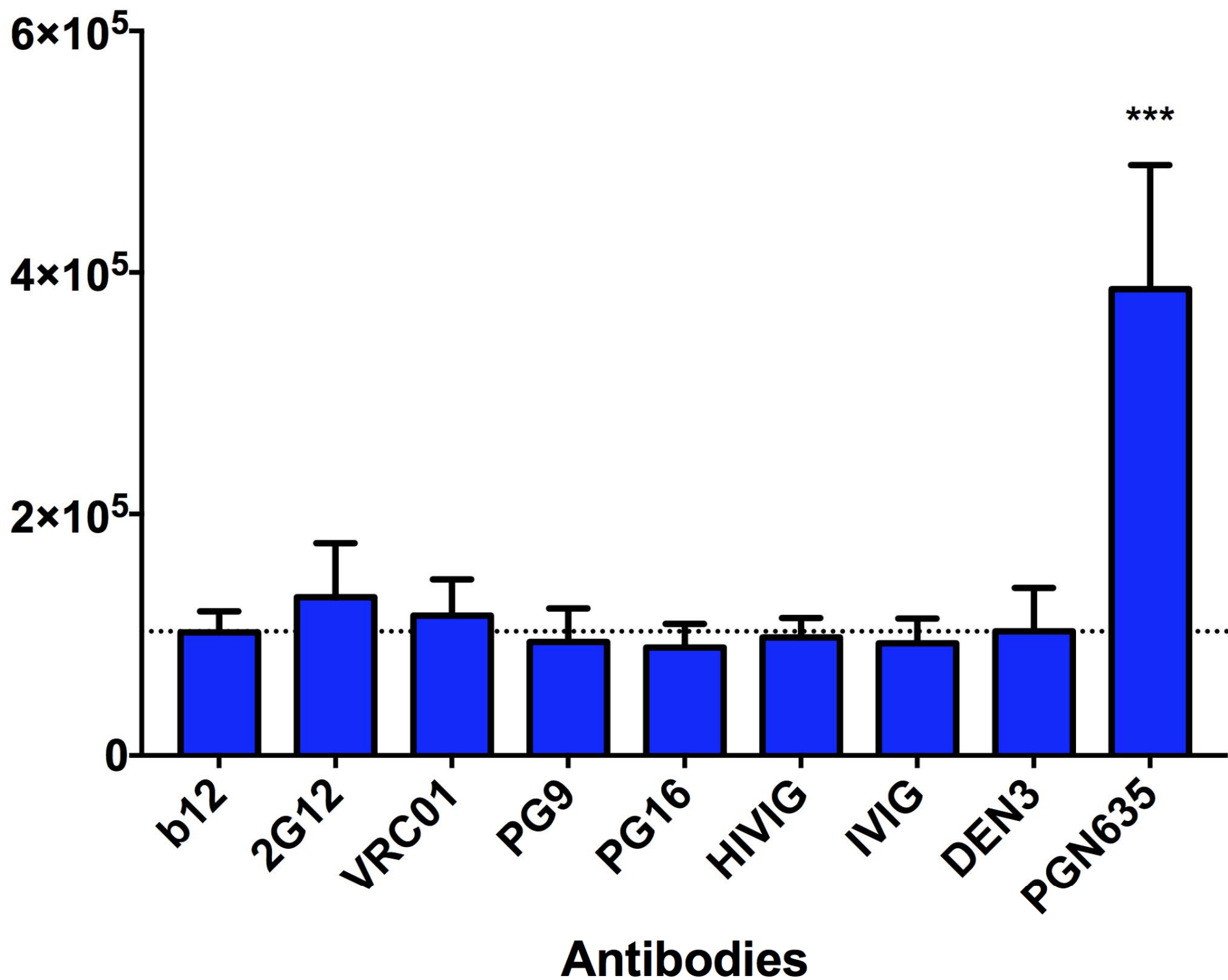

Supplement: S3 Fig — Uptake of HIV-1JRFl virions was measured in U937 cells by quantitative RT-PCR. All mAbs were tested at a concentration of 0.02 mg/mL, and the polyclonal antibodies HIVIG and IVIG at a concentration of 0.08 mg/mL. PGN635 was used as a positive control. Data are reported as viral RNA copy numbers per 25,000 cells. One-way ANOVA was used to analyze virus uptake in the presence of antibody compared to the no antibody control (dotted line). P-values are indicated with an asterisk: * p ≤ 0.05; ** p ≤ 0.01, and *** p ≤ 0.001. All phagocytosis experiments were performed in triplicate and repeated at least three times. Data are reported as means + SEM. (PDF) [file ppat.1006793.s003.pdf]

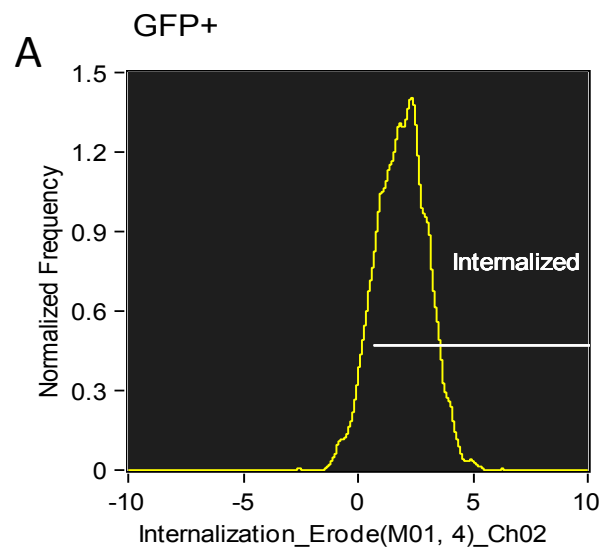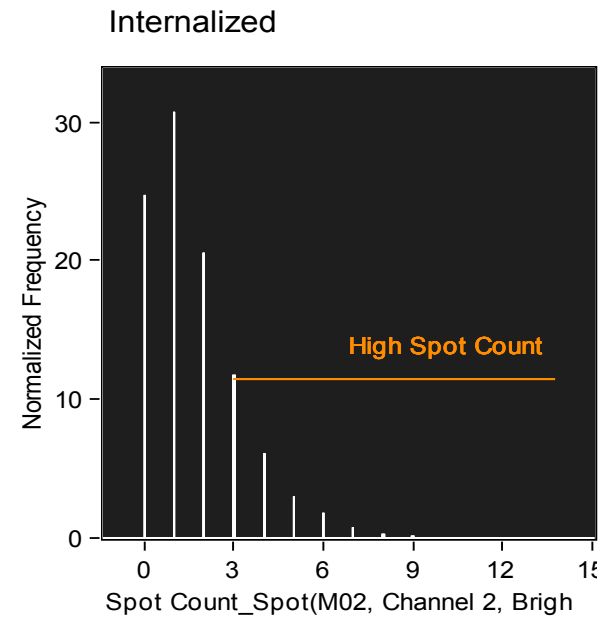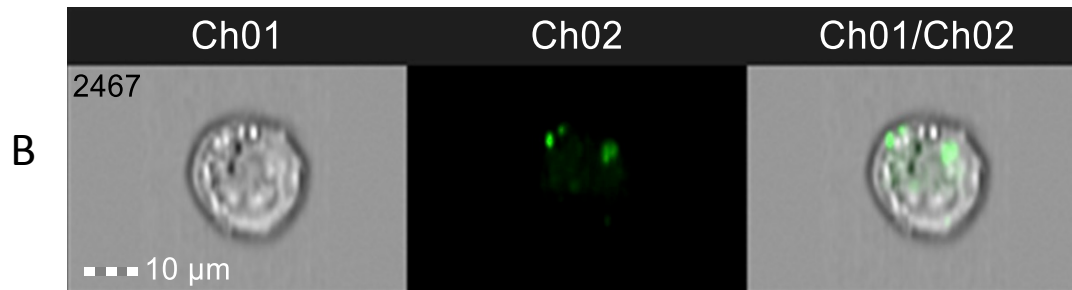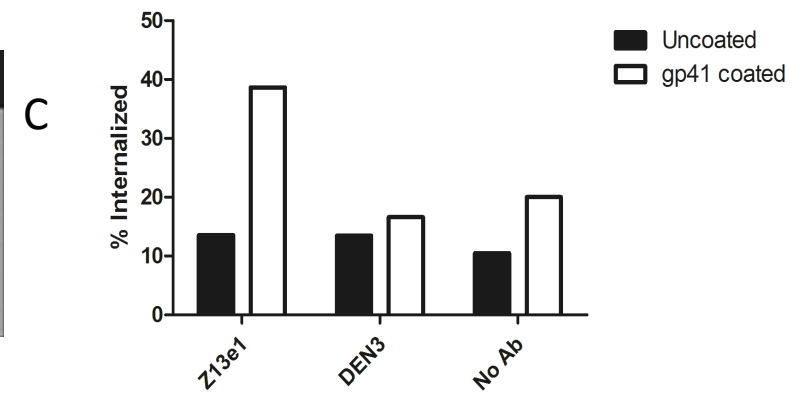

Supplement: S5 Fig — Image Stream was used to quantify Z13e1-opsonized or unopsonized virus internalized by THP-1 effector cells. Internalization of positive events was measured by applying the ImageStream IDEAS Internalization and Spot Wizard algorithms, which defined the internal area as a mask of erosion of 4 pixels into the brightfield perimeter of the cell. According to the protocol outlined in [41], an internalization score of 0.3 and a spot count of 3 were used to identify internalized virions and exclude surface-bound virions and background fluorescence. The gating strategy (A), a representative image (B), and percent of cells with internalized virus compared to the total number of focused, single cells (C) are shown. More than 10,000 images per condition were collected. (PDF) [file ppat.1006793.s005.pdf]

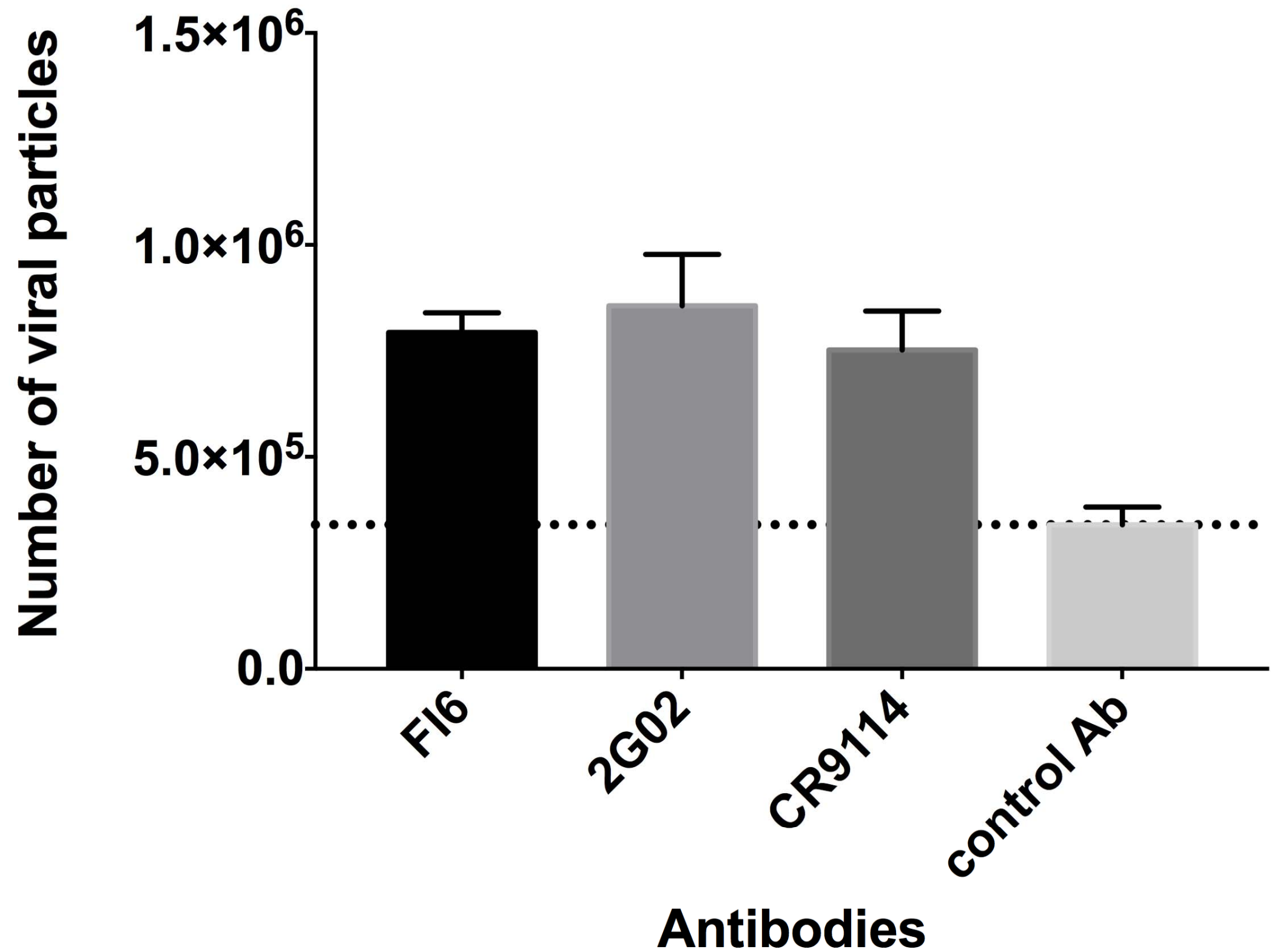

Supplement: S6 Fig — Internalization of influenza virus was measured by qPCR of lysed THP-1 cells following a 60- to 80-minute period of exposure of cells to virus opsonized with anti-hemagglutinin mAbs FI6, 2G02, and CR9114. All mAbs were tested at a concentration of 0.01 mg/mL. Data represent means + SEM of duplicate independent experiments, each performed in triplicate. (PDF) [file ppat.1006793.s006.pdf]

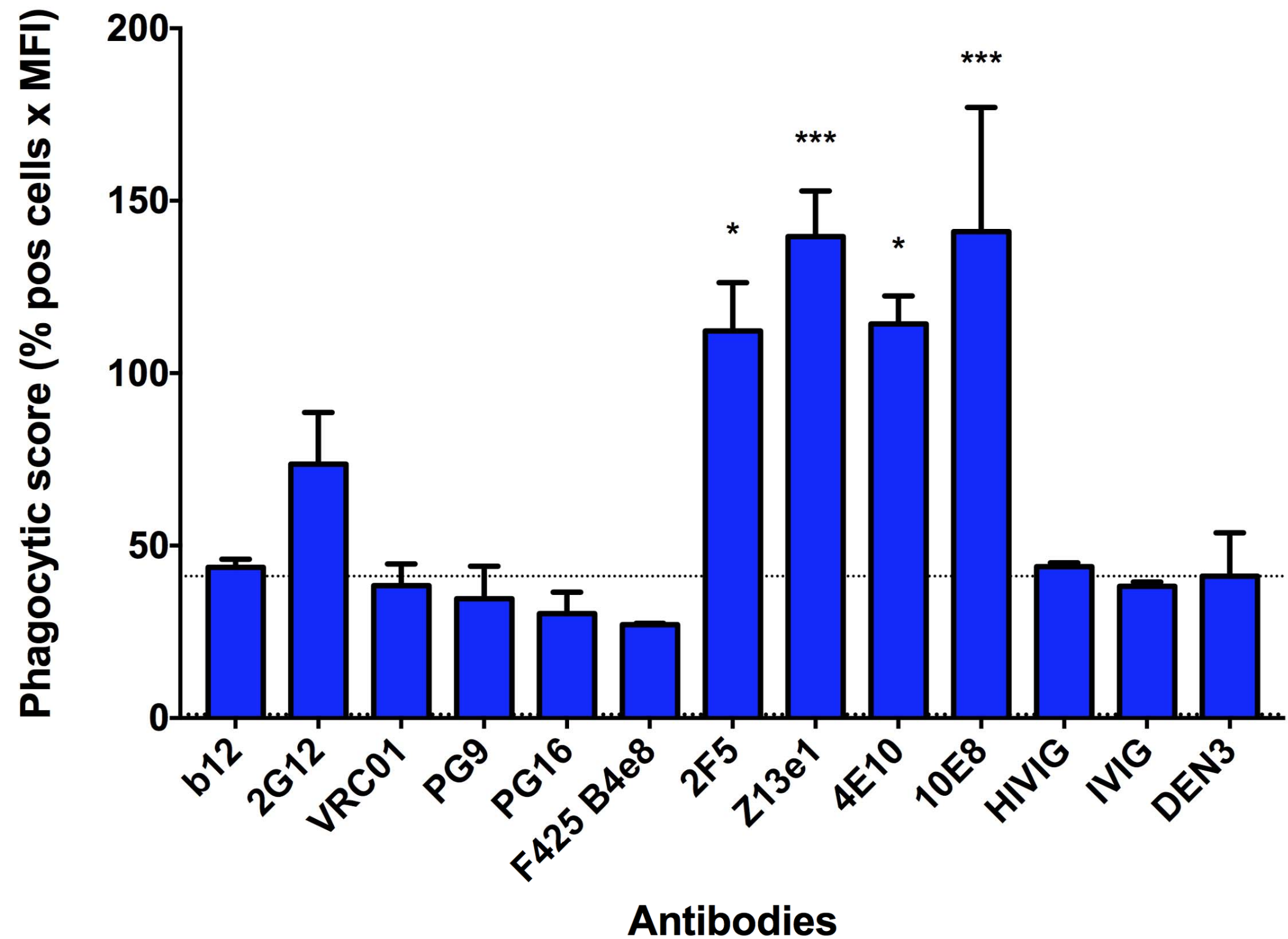

Supplement: S7 Fig — To induce aggregation, HIV-1iGFP/JR-FL virions were opsonized with HIV-specific antibodies and subsequently incubated with a goat F(ab’)2 anti-human F(ab’)2 antibody (0.05 mg/mL) prior to being fed to U937 cells. All mAbs were tested at a concentration of 0.05 mg/mL and the polyclonal antibodies HIVIG and IVIG at a concentration of 0.2 mg/mL. One-way ANOVA was used to analyze virus uptake in the presence of antibody compared to the DEN3 control mAb (dotted line). P-values are indicated with an asterisk: * p ≤ 0.05; ** p ≤ 0.01, and *** p ≤ 0.001. Experiments were performed in triplicate and repeated at least twice. Data are reported as means + SEM. (PDF) [file ppat.1006793.s007.pdf]

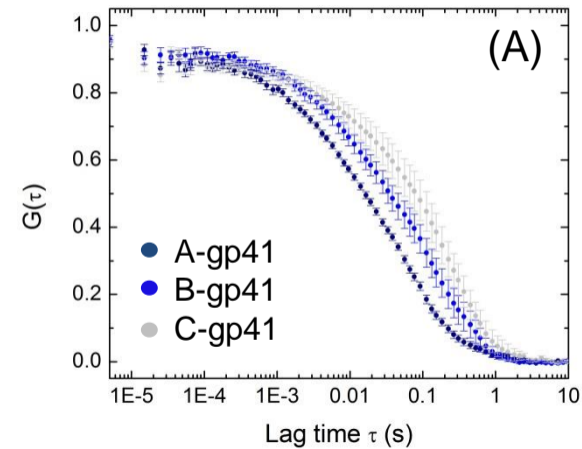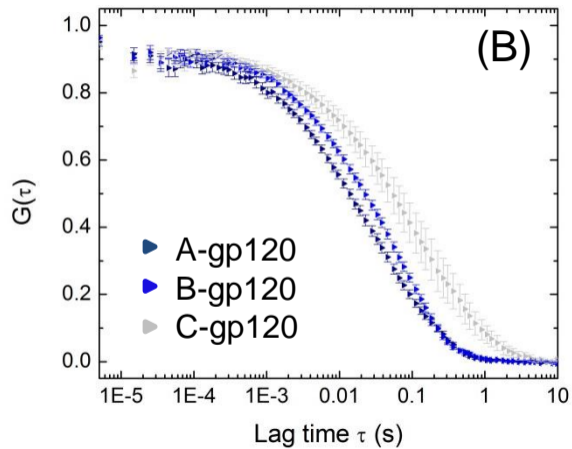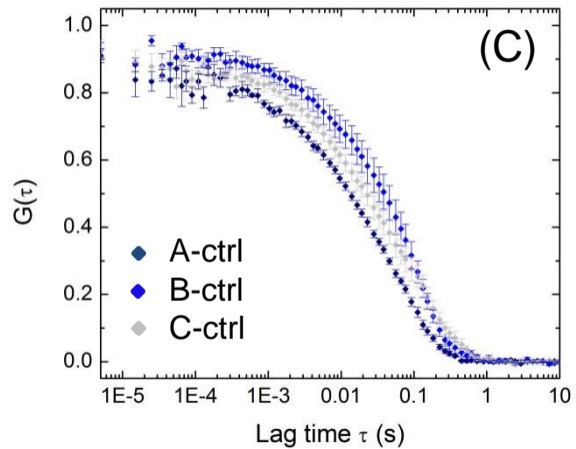

Supplement: S8 Fig — Normalized experimental average ACFs recovered for group A, B, and C virus opsonized with anti-gp41 antibody (A), anti-gp120 antibody (B) or unopsonized (C). Data are reported as mean ± SEM. Only half of the correlation data points are reported for the sake of visual clarity. (PDF) [file ppat.1006793.s008.pdf]

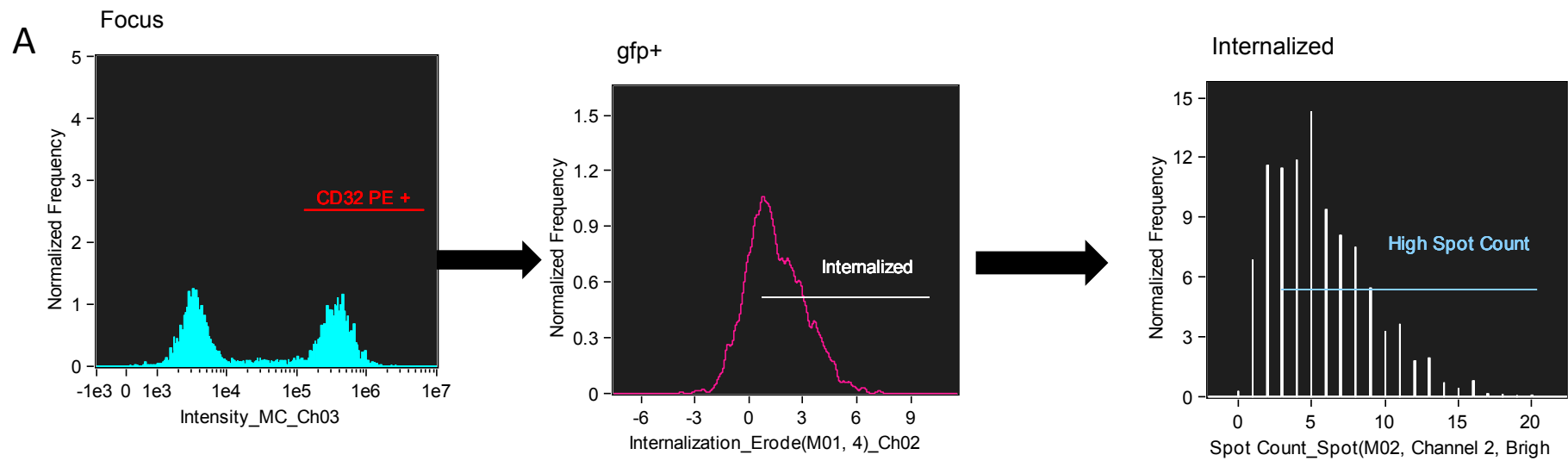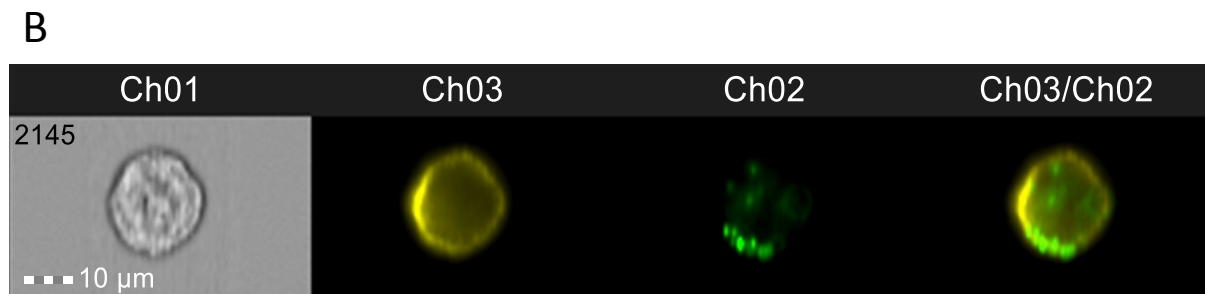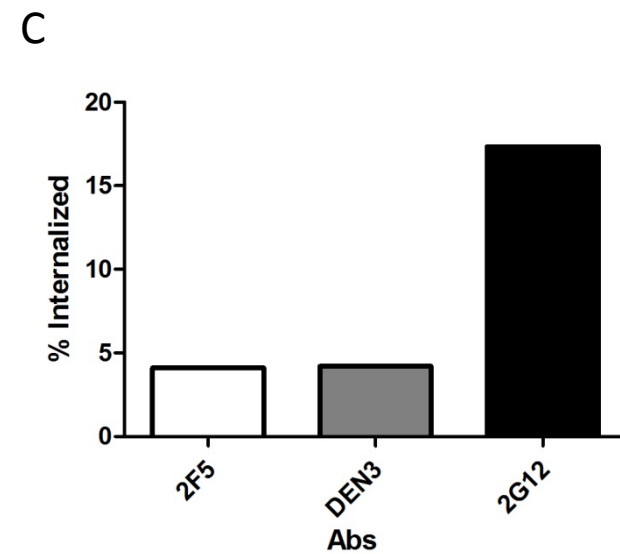

Supplement: S9 Fig — CEM.NKr-CCR5 decorated with virus at 4°C and opsonized with 2G12 or control antibodies were incubated with THP-1 effector cells. THP-1 cells were identified by PE-conjugated anti-CD32 antibody staining. Using ImageStream cytometry, internalization of positive events was measured as described in S5 Fig. The gating strategy (A), a representative image (B), and the percent of cells with internalized virus compared to the total number of focused, single cells (C) are shown. (PDF) [file ppat.1006793.s009.pdf]

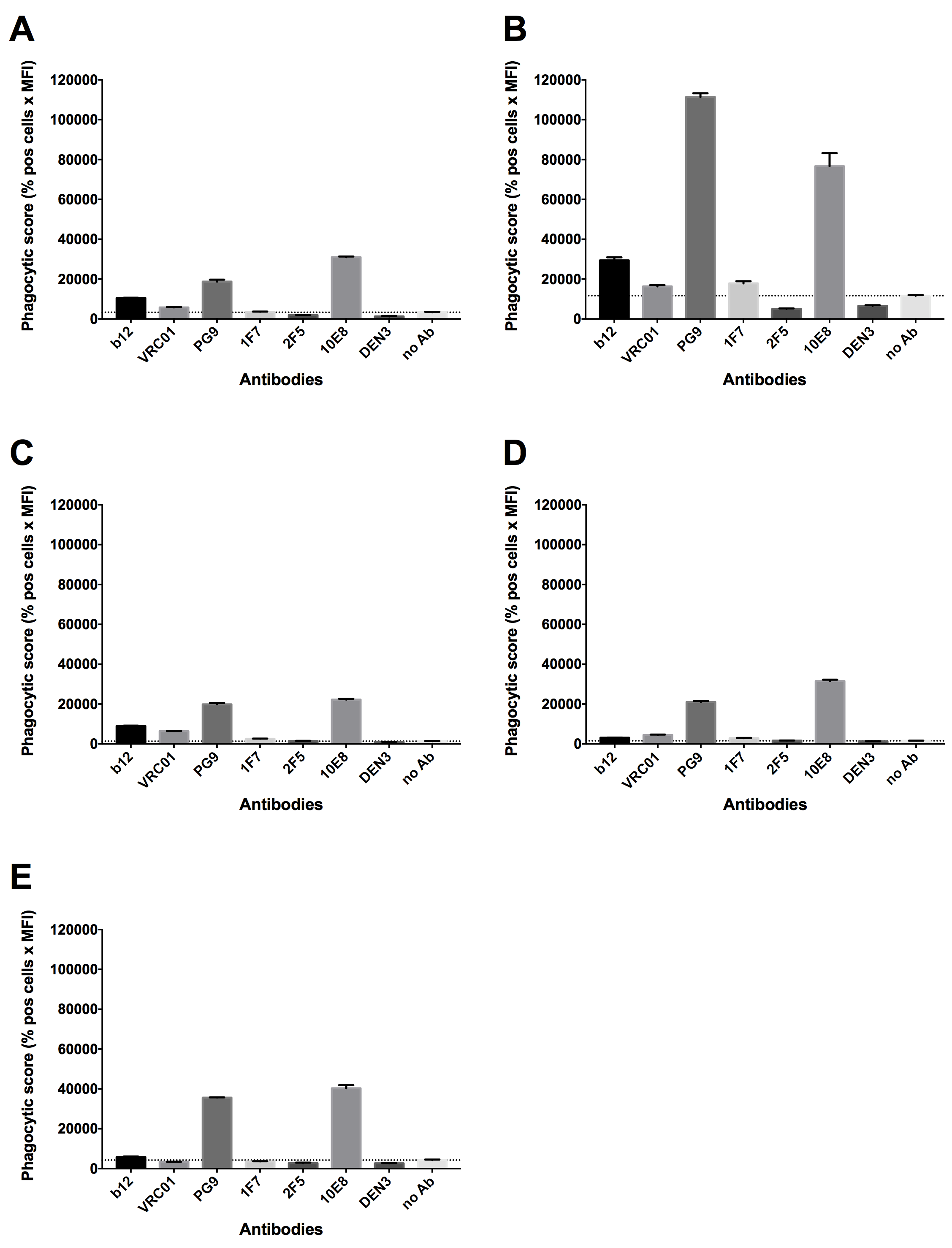

Supplement: S10 Fig — Prior to measuring phagocytosis by THP-1 cells, HIV-1 molecular clones or pseudotyped HIV-1VSV-g (control) were stained with DiD for 30 min at 37°C and subsequently washed with medium. Flow cytometry revealed high levels of ADP in the case of PG9 and 10E8 for all HIV-1 isolates including HIV-1SF162 (A), HIV-1HxB2 (B), HIV-1JR-CSF (C), and HIV-1ADA (D), as well as for the HIV-1VSV-g pseudotyped negative control (E). Antibodies were tested at a concentration of 0.05 mg/mL and the polyclonal antibody HIVIG at a concentration of 0.2 mg/mL. The phagocytic score of the no antibody control is indicated by the dotted line. Experiments were performed in triplicate and were repeated at least twice with similar results. (TIFF) [file ppat.1006793.s010.tiff]

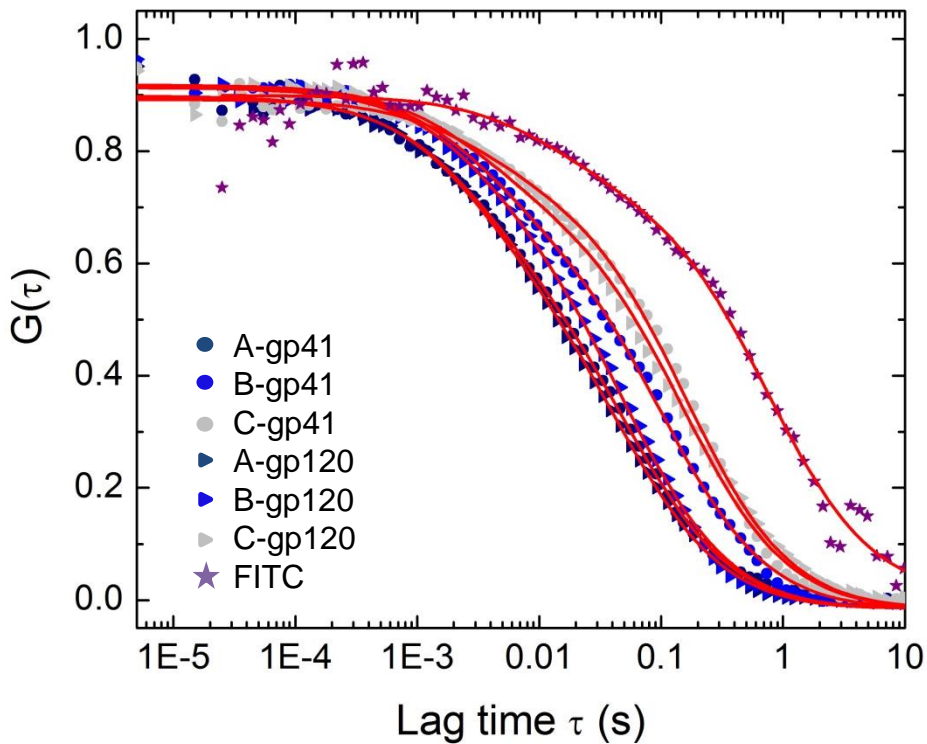

Supplement: S11 Fig — Normalized experimental average ACF recovered for FITC-labeled virions freely diffusing in 2% PAF solution is shown. The fit (red continuous line) was performed to eq. S. 4 (S1 Text) with best-fit parameters D1 = 1.5 ± 0.5 μm2/s, D2 = 0.029 ± 0.003 μm2/s, G01 = 0.17 ± 0.02 and G02 = 0.72 ± 0.02 μm2/s. The corresponding hydrodynamic radii were computed based on the Stokes-Einstein equation as R1 = 140±47 nm and R2 = 7262±751 nm. Normalized experimental average ACFs measured for opsonized or unopsonized group A, B, and C virus, together with their global fit to eq. S. 4 (S1 Text), are also reported for comparison. Only half of the correlation data points are shown for the sake of visual clarity. (PDF) [file ppat.1006793.s011.pdf]

**A**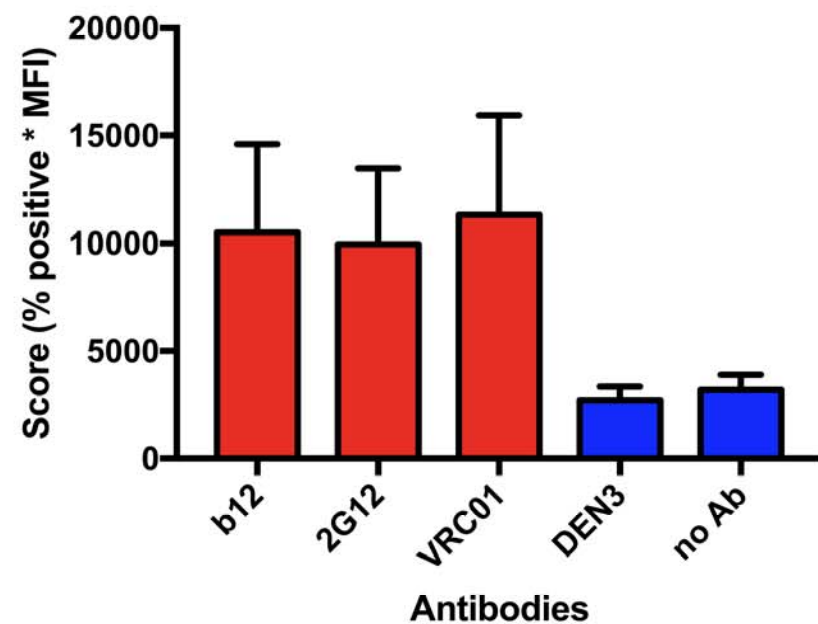**B**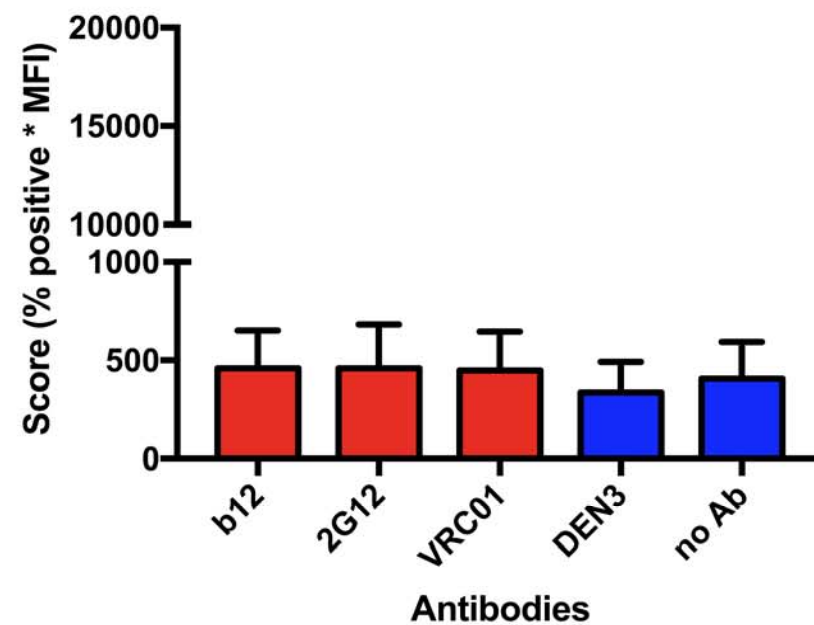**C**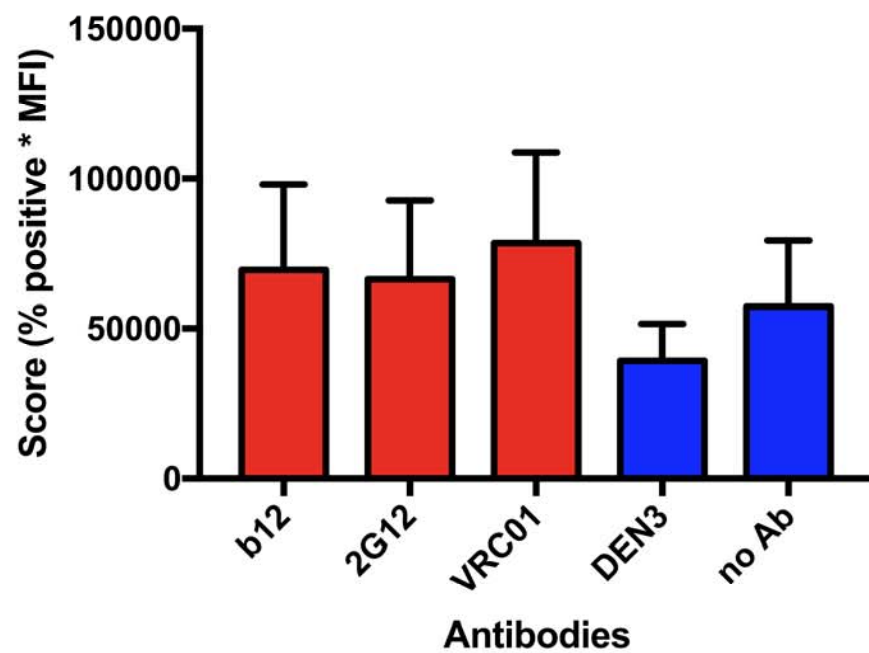**D**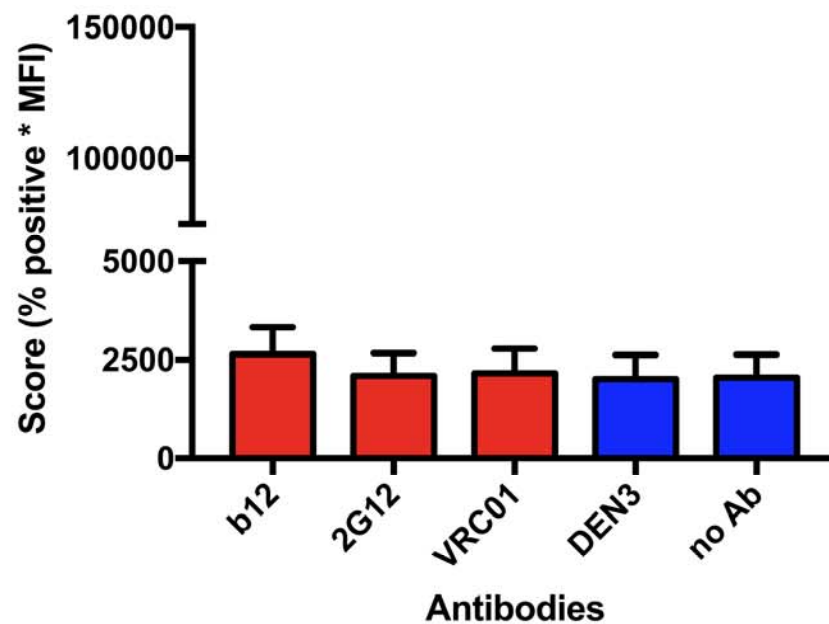

Supplement: S12 Fig — HIV-1iGFP/JR-FL at 10 ng (A and B) or 50 ng (C and D) of p24 was opsonized with b12, 2G12, or VRC01 or with DEN3 (unopsonized). Virus-mAb preparations were then either spun in a microfuge at 1200 g for 1 hour with THP-1 cells at 4°C to avoid internalization (A and C) or left unspun as in all previous experiments (B and D). Cells were then fixed on ice, and flow cytometry was used to quantify THP-1 cells with virus on their surface. Shown are the means + SEM of five independent experiments. When spinning was employed, each HIV-specific mAb resulted in significantly greater surface binding of HIV-1iGFP/JR-FL than did the DEN3 control mAb (p < 0.05). In some cases, there were significant differences between opsonized and unopsonized virus in the absence of spinning (b12 and VRC01 at 10 ng of p24 and 2G12 at 50 ng of p24), whereas there were no significant differences (p > 0.05) with 2G12 at 10 ng or with b12 and VRC01 at 50 ng of p24. (PDF) [file ppat.1006793.s012.pdf]
