# Supplementary material for: Human immunodeficiency virus type-1 (HIV-1) evades antibody-dependent phagocytosis
Source: PLoS Pathog. 2017 Dec 27;13(12):e1006793. doi: 10.1371/journal.ppat.1006793 (PMC5760106; doi:10.1371/journal.ppat.1006793)
Supplement: S4 Fig — MPER peptide captured with NeutrAvidin (A) or recombinant HIV-1MN gp41 protein captured with D50 antibody (B) was used to measure antibody binding by ELISA. Data represent the means from two independent experiments in duplicate. (PDF) [file ppat.1006793.s004.pdf]

A

		MPER peptide 09129											
	**Antibody (µg/mL)**	b12	2G12	VRC01	PG9	PG16	F425	2F5	Z13e1	4E10	10E8	HIVIG	DEN3
OD450	0.4	0.000	0.000	0.000	0.000	0.000	0.000	2.529	2.424	1.091	0.073	0.000	0.000
	2	0.000	0.000	0.000	0.000	0.000	0.000	2.827	2.993	1.298	0.198	0.000	0.000
	10	0.000	0.000	0.000	0.000	0.000	0.000	3.003	3.050	1.994	0.374	0.000	0.000
	50	0.000	0.048	0.000	0.000	0.000	0.000	2.998	3.146	2.852	0.562	0.000	0.000

B

		HIV-1 MN gp41											
	**Antibody (µg/mL)**	b12	2G12	VRC01	PG9	PG16	F425	2F5	Z13e1	4E10	10E8	HIVIG	DEN3
OD450	0.4	0.000	0.000	0.000	0.000	0.000	0.000	0.745	2.468	0.038	0.000	0.206	0.000
	2	0.000	0.000	0.000	0.000	0.000	0.000	1.423	2.929	0.202	0.000	0.856	0.000
	10	0.016	0.007	0.000	0.013	0.000	0.017	1.977	3.181	0.714	0.015	2.186	0.000
	50	0.133	0.070	0.006	0.086	0.004	0.098	2.479	3.279	1.915	0.084	3.033	0.001
