# Supplementary material for: Human immunodeficiency virus type-1 (HIV-1) evades antibody-dependent phagocytosis
Source: PLoS Pathog. 2017 Dec 27;13(12):e1006793. doi: 10.1371/journal.ppat.1006793 (PMC5760106; doi:10.1371/journal.ppat.1006793)
Supplement: S1 Table — The diffusion coefficient D1 of the non-aggregate population and the correlation offset G∞ were treated as shared parameters for the global fit of the nine ACFs. Values reported as minimum and maximum (left and right columns, respectively) were obtained by the FCS rigorous error analysis (i.e., they were selected by evaluating all the possible parameter combinations compatible with a predefined maximum variation [one standard deviation] of the ACFs global-fit chi-square value). (DOCX) [file ppat.1006793.s013.docx]

**S1 Table.** Best-fit parameters (central column) recovered by the global fit of the average ACFs of groups A, B, or C virus (opsonized with anti-gp41, anti-gp120 antibody, or unopsonized as indicated; ACFs are reported in **Fig 5** and **S8 Fig**). The diffusion coefficient D_1_ of the non-aggregate population and the correlation offset G_∞_ were treated as shared parameters for the global fit of the nine ACFs. Values reported as minimum and maximum (left and right columns, respectively) were obtained by the FCS rigorous error analysis (i.e., they were selected by evaluating all the possible parameter combinations compatible with a predefined maximum variation [one standard deviation] of the ACFs global-fit chi-square value).

|  | min D_2_ (µm^2^/s) | best-fit D_2_ (µm^2^/s) | max D_2_ (µm^2^/s) |
| --- | --- | --- | --- |
| A-anti-gp41 | 0.31 | 0.39 | 0.51 |
| A-anti-gp120 | 0.33 | 0.45 | 0.57 |
| A-unopsonized | 0.45 | 0.57 | 0.69 |
| B-anti-gp41 | 0.21 | 0.25 | 0.31 |
| B-anti-gp120 | 0.37 | 0.49 | 0.61 |
| B-unopsonized | 0.41 | 0.48 | 0.56 |
| C-anti-gp41 | 0.13 | 0.15 | 0.17 |
| C-anti-gp120 | 0.13 | 0.16 | 0.19 |
| C-unopsonized | 0.31 | 0.37 | 0.45 |
|  | min D_1_ (µm^2^/s) | best-fit D_1_ (µm^2^/s) | max D_1_ (µm^2^/s) |
| All | 5.0 | 7.0 | 9.5 |
|  | min G0_1_ | best-fit G0_1_ | max G0_1_ |
| A-anti-gp41 | 0.25 | 0.32 | 0.40 |
| A-anti-gp120 | 0.24 | 0.33 | 0.41 |
| A-unopsonized | 0.17 | 0.23 | 0.31 |
| B-anti-gp41 | 0.21 | 0.27 | 0.33 |
| B-anti-gp120 | 0.19 | 0.26 | 0.34 |
| B-unopsonized | 0.05 | 0.12 | 0.18 |
| C-anti-gp41 | 0.13 | 0.16 | 0.20 |
| C-anti-gp120 | 0.18 | 0.23 | 0.27 |
| C-unopsonized | 0.17 | 0.23 | 0.30 |
|  | min G0_2_ | best-fit G0_2_ | max G0_2_ |
| A-anti-gp41 | 0.52 | 0.59 | 0.66 |
| A-anti-gp120 | 0.51 | 0.59 | 0.67 |
| A-unopsonized | 0.55 | 0.63 | 0.69 |
| B-anti-gp41 | 0.60 | 0.66 | 0.72 |
| B-anti-gp120 | 0.59 | 0.67 | 0.73 |
| B-unopsonized | 0.74 | 0.80 | 0.86 |
| C-anti-gp41 | 0.72 | 0.75 | 0.78 |
| C-anti-gp120 | 0.67 | 0.71 | 0.75 |
| C-unopsonized | 0.60 | 0.66 | 0.72 |
|  | min G_∞_ | best-fit G_∞_ | max G_∞_ |
| All | -0.025 | -0.016 | -0.005 |
