# Supplementary material for: Human immunodeficiency virus type-1 (HIV-1) evades antibody-dependent phagocytosis
Source: PLoS Pathog. 2017 Dec 27;13(12):e1006793. doi: 10.1371/journal.ppat.1006793 (PMC5760106; doi:10.1371/journal.ppat.1006793)
Supplement: S2 Table — Radii are reported in Fig 5C). * p ≤ 0.0332; ** p ≤ 0.0021, *** p ≤ 0.0002 and **** p ≤ 0.0001). (DOCX) [file ppat.1006793.s014.docx]

**S2 Table.** One-way ANOVA followed by Tukey’s multiple-comparisons test was performed on the hydrodynamic radii of the aggregate population recovered for all combinations of virus groups and antibody opsonization groups. Radii are reported in **Fig 5C**). * *p* ≤ 0.0332; ** *p* ≤ 0.0021, *** *p* ≤ 0.0002 and **** *p* ≤ 0.0001).

|  | significance | p value |  | significance | p value |
| --- | --- | --- | --- | --- | --- |
| A-anti-gp41, A-anti-gp120 | ns | 0.1322 | A-unopsonized, C-anti-gp41 | **** | <0.0001 |
| A-anti-gp41, A-unopsonized | *** | 0.0003 | A- unopsonized, C-anti-gp120 | **** | <0.0001 |
| A-anti-gp41, B-anti-gp41 | **** | <0.0001 | A- unopsonized, C- unoponized | *** | 0.0003 |
| A-anti-gp41, B-anti-gp120 | *** | 0.0009 | B- anti-gp41, B- anti-gp120 | **** | <0.0001 |
| A-anti-gp41, B-unopsonized | ns | 0.0995 | B- anti-gp41, B- unopsonized | **** | <0.0001 |
| A-anti-gp41, C-anti-gp41 | **** | <0.0001 | B- anti-gp41, C- anti-gp41 | **** | <0.0001 |
| A-anti-gp41, C-anti-gp120 | **** | <0.0001 | B- anti-gp41, C- anti-gp120 | **** | <0.0001 |
| A-anti-gp41, C-unopsonized | ns | 0.9904 | B- anti-gp41, C- unopsonized | **** | <0.0001 |
| A-anti-gp120, A-unopsonized | * | 0.0417 | B- anti-gp120, B- unopsonized | ns | >0.9999 |
| A-anti-gp120, B-anti-gp41 | **** | <0.0001 | B- anti-gp120, C- anti-gp41 | **** | <0.0001 |
| A-anti-gp120, B-anti-gp120 | ns | 0.4587 | B- anti-gp120, C- anti-gp120 | **** | <0.0001 |
| A-anti-gp120, B-unopsonized | ns | 0.9785 | B- anti-gp120, C- unopsonized | ** | 0.0024 |
| A-anti-gp120, C-anti-gp41 | **** | <0.0001 | B- unopsonized, C- anti-gp41 | **** | <0.0001 |
| A-anti-gp120, C-anti-gp120 | **** | <0.0001 | B- unopsonized, C- anti-gp120 | **** | <0.0001 |
| A-gp120, C-ctrl | ns | 0.0902 | B- unopsonized, C- unopsonized | ns | 0.0538 |
