# Supplementary material for: Human immunodeficiency virus type-1 (HIV-1) evades antibody-dependent phagocytosis
Source: PLoS Pathog. 2017 Dec 27;13(12):e1006793. doi: 10.1371/journal.ppat.1006793 (PMC5760106; doi:10.1371/journal.ppat.1006793)
Supplement: S1 Text — (DOCX) [file ppat.1006793.s015.docx]

**S1 Text**

**FCS THEORY**

The theory of fluorescence correlation spectroscopy (FCS) has been described and reviewed previously [**Magde 1972, Elson 1974, Digman 2011, Elson 2011**]. We just summarize here the basic principles.

The raw data in FCS experiments consist in a temporal intensity trace of the fluorescence signal emitted by a highly diluted (1-100 nM concentration) steady-state sample in a typical µm^3^ open volume, defined by a diffraction-limited excitation laser beam and confocal detection optics. By affecting the fluorophores quantum yield and/or the number of molecules inside the excitation volume, processes such as molecular translational and rotational diffusion, binding and unbinding events, photo-physical or chemical reactions induce fluorescence fluctuations over time. Such spontaneous fluctuations dissipate on a characteristic time scale assigned by the underlying reaction constants and transport coefficients, so that these parameters can in principle be quantified by the statistical analysis of the recorded fluorescence time trace. To this aim, a normalized temporal Auto-Correlation Function (ACF) of the detected fluorescence signal is defined and computed as [**Magde 1972, Elson 1974, Digman 2011, Elson 2011**]

$$G\left( \tau\right)=\frac{\left\langle\delta F\left( t \right)\delta F(t+\tau) \right\rangle_{t}}{\left\langle F(t) \right\rangle_{t}^{2}}=\frac{\left\langle F\left( t \right)F(t+\tau) \right\rangle_{t}}{\left\langle F(t) \right\rangle_{t}^{2}}-1 (S.1)$$

τ is the correlation lag time and F(t) and F(t+τ) are the fluorescence intensities at time points t and t+τ. Fluorescence fluctuations are defined as $\delta F\left( t \right)=F\left( t \right)-\left\langle F(t) \right\rangle_{t}$ and angular brackets $\left\langle\ldots\right\rangle_{t}$ denote a time average over the total acquisition time T. T is required to be at least two orders of magnitude larger than the correlation function decay time in order for the fluctuations to be sampled accurately.

G(τ) is a decaying function of the lag time τ, and its explicit analytical expression depends on the source of the fluorescence fluctuations and on the shape of the excitation volume. We are interested here in a homogeneous sample undergoing isotropic three-dimensional Brownian diffusion with diffusion coefficient D through a confocal excitation volume, modeled by a 3D Gaussian Point-Spread-Function (PSF). In this case G(τ) takes the form [**Elson 2011, Meseth 1999**]

$$G\left( \tau\right)=G0\frac{1}{1+\frac{4D\tau}{\omega_{0}^{2}}}\frac{1}{\sqrt{1+\zeta^{2}\frac{4D\tau}{\omega_{0}^{2}}}}+G_{\infty}\equiv f(\tau,G0,D) (S.2)$$

G0=γ/N in eq. (S.2) is the correlation amplitude at zero lag time: it is inversely proportional to the average number N of fluorescent molecules inside the excitation volume, and directly proportional to a factor γ correcting for the shape of the excitation point-spread-function (γ=0.35 for a 3D Gaussian PSF). G_∞_ is the ideally-zero limiting value of the correlation function for $\tau\to+\infty$, while ζ is the excitation volume form factor. It is defined as the ratio of the radial and axial excitation laser beam waists (ζ=ω_0_/ω_z_) and typically ζ=1/5 for a confocal setup. The more compact notation $G\left( \tau\right)\equiv f(\tau,G0,D)$ in eq. (S.2) implies the instrumental parameters ω_0_ and ζ, and highlights the dependence of the ACF on the particles transport properties and concentration via the diffusion coefficient D and the G_0_ correlation amplitude.

In order to recover D and G_0_, a non-linear least-squares fit of the experimental ACF to eq. (S.2) is performed. While D, G_0_ and the G_∞_ offset are treated as variable parameters in the fitting procedure, both ω_0_ and ζ are fixed to their previously-calibrated values (they can be measured either by collecting the experimental ACF of a reference sample of known diffusion coefficient, or by confocal imaging of sub-resolved fluorescent objects). Once the best-fit diffusion coefficient D has been determined, the molecules hydrodynamic radius R is finally recovered, under a spherical shape approximation, via the well-known Stokes-Einstein’s equation:

$$R=\frac{KT}{6\pi\eta D} (S.3)$$

T, K and $\eta$ in eq. (S.3) denote the system temperature, Boltzmann’s constant and the solution viscosity, respectively. Room temperature T=298 K and water viscosity were assumed in the present work for our 2% PAF solutions **[Winkelman 2000]**.

The shape and the analytical expression of the ACF change in the presence of molecular aggregation **[Meseth 1999]**. For a non-aggregated population with average hydrodynamic radius R_1_ (single virions, in our case) and larger aggregates with average radius R_2_ simultaneously undergoing isotropic 3D Brownian diffusion, the FCS auto-correlation function turns into the linear combination of two single-component ACFs of the type we have previously introduced with eq. (S.2):

$$G\left( \tau\right)=f\left( \tau,{G0}_{1},D_{1} \right)+f\left( \tau,{G0}_{2},D_{2} \right) (S.4)$$

D_1_ and D_2_ in eq. (S.4) are the diffusion coefficients of the two populations. As in the case of the single-component diffusive model (eq. S.2), the measurement of the diffusion coefficients by the non-linear least-squares fit of the experimental ACF to eq. (S.4) allows retrieving the estimates of the average hydrodynamic radii R_1_ and R_2_ of the two molecular species via the Stokes-Einstein’s equation. Correlation amplitudes G0_i=1,2_ in eq. (S.4) contain the dependence on the concentration and fluorescence quantum yield of the two diffusive components. Denoted with N_i=1,2_ the average number of molecules in the excitation volume for species *i*,

$${G0}_{i}=\frac{\gamma\phi_{i}^{2}N_{i}}{\left( \sum_{k=1}^{2} \phi_{k}N_{k} \right)^{2}} (S.5)$$

φ_i=1,2_ in eq. (S.5) defines the product of the absorbance, fluorescence quantum yield and experimental fluorescence collection efficiency for the molecules of species *i* **[Meseth1999]**. We remark that such a dependence of the correlation fractions G0_1_ and G0_2_ on the quantum yields of the two diffusing populations prevents the quantification of their absolute abundance (i.e., N_1_ and N_2_).

**Magde D, Elson E, Webb WW. Thermodynamic fluctuations in a reacting system: measurement by fluorescence correlation spectroscopy. 1972. Phys Rev Lett 29:705–8**

**Elson EL, Magde D. Fluorescence correlation spectroscopy. I. Conceptual basis and theory. 1974. Biopolymers. 13:1-27**

**Digman MA, Gratton E. Lessons in fluctuation correlation spectroscopy. 2011. Annu Rev Phys Chem. 62:645–68**

**Elson EL. 2011. Fluorescence correlation spectroscopy: past, present, future. Biophys J. 101:2855-2870**

**Meseth U, Wohland T, Rigler R, Vogel H. Resolution of fluorescence correlation measurements. 1999. Biophys J. 76:1619-1631**

**Winkelman JGM, Beenackers, ACM. Correlations for the density and viscosity of aqueous formaldehyde solutions. 2000. Ind Eng Chem Res. 39:557-562**
